# 3D Cancer Models: Depicting Cellular Crosstalk within the Tumour Microenvironment

**DOI:** 10.3390/cancers13184610

**Published:** 2021-09-14

**Authors:** Teresa Franchi-Mendes, Rodrigo Eduardo, Giacomo Domenici, Catarina Brito

**Affiliations:** 1 iBET—Instituto de Biologia Experimental e Tecnológica, Apartado 12, 2781-901 Oeiras, Portugal; mtfmendes@gmail.com (T.F.-M.); rodrigomeduardo33@gmail.com (R.E.); giacomo.domenici@ibet.pt (G.D.); 2 Instituto de Tecnologia Química e Biológica António Xavier, Universidade Nova de Lisboa, Av. da República, 2780-157 Oeiras, Portugal; 3 The Discoveries Centre for Regenerative and Precision Medicine, Lisbon Campus, Av. da República, 2780-157 Oeiras, Portugal

**Keywords:** 3D cell models, tumour microenvironment, heterotypic interactions, cell communication, immune infiltrate, cancer-associated fibroblasts, tumour-associated endothelial cells, tumour spheroids, hydrogels

## Abstract

**Simple Summary:**

The tumour microenvironment is composed of multiple non-cancerous cells that communicate with the tumour cells, influencing their behaviour and impacting the progression of the disease and the response to therapy. To better understand the disease and try to predict the response of patients to therapy, there has been an effort to develop experimental strategies that could represent this complex human tumour microenvironment in a dish (in vitro). In this review, we describe the importance of each cell type and review the in vitro approaches recently developed for cultivating together the different cell types (co-culture) in a three-dimensional configuration to better represent the architecture of the tumour and cell interactions (3D models). We describe and compare the different studies and outline perspectives on the 3D modelling strategies and their potential impact in cancer research and anticancer drug discovery.

**Abstract:**

The tumour microenvironment plays a critical role in tumour progression and drug resistance processes. Non-malignant cell players, such as fibroblasts, endothelial cells, immune cells and others, interact with each other and with the tumour cells, shaping the disease. Though the role of each cell type and cell communication mechanisms have been progressively studied, the complexity of this cellular network and its role in disease mechanism and therapeutic response are still being unveiled. Animal models have been mainly used, as they can represent systemic interactions and conditions, though they face recognized limitations in translational potential due to interspecies differences. In vitro 3D cancer models can surpass these limitations, by incorporating human cells, including patient-derived ones, and allowing a range of experimental designs with precise control of each tumour microenvironment element. We summarize the role of each tumour microenvironment component and review studies proposing 3D co-culture strategies of tumour cells and non-malignant cell components. Moreover, we discuss the potential of these modelling approaches to uncover potential therapeutic targets in the tumour microenvironment and assess therapeutic efficacy, current bottlenecks and perspectives.

## 1. Introduction

Recapitulative disease models are important experimental tools, particularly in the oncology field in which new drugs fail in clinical trials more than in any other area [1]. During the drug development pipeline, more than 95% of the anticancer agents will not reach the market [1]. Therefore, ongoing research on experimental cancer modelling aims at achieving better predictions of drug efficacy by increasing the translational potential of the models employed [2]. 

Tumours are composed of heterogeneous populations of tumour cells, as well as non-malignant cells and non-cellular elements, such as extracellular matrix (ECM) and soluble factors secreted by the different cell types [3]. The non-malignant cellular components and non-cellular elements constitute what is defined as the tumour microenvironment (TME). The role of the TME in tumorigenesis, tumour progression, invasion and metastasis has been acknowledged over recent decades and is today unquestionable [4,5,6,7]. Moreover, the TME has been increasingly implicated in the modulation of drug response and resistance [8,9,10,11]. Therefore, the use of therapeutic agents targeting TME-mediated signalling or TME composition has been proposed [3,4,12], such as the drugs that inhibit matrix metalloproteinase (MMP) activity [12], disrupt angiogenesis [13] or immunomodulators (e.g., immune checkpoint inhibitors) [14].

The cellular elements of the TME may vary within different cancers and consequently the ECM and other non-cellular components. In solid tumours, non-malignant cells can be recruited locally (tissue-resident) and systemically [8,15] and comprise mainly fibroblasts, endothelial cells (EC), and innate and adaptive immune cells [8,16]. Mesenchymal stromal cells (MSC), adipocytes and other bone marrow-derived cells have also been reported [3]. In addition to direct cell–cell contacts between tumour cells and the different TME cell types and amongst the latter, the main TME mediators are soluble factors (such as cytokines, chemokines, proteases, and other enzymes involved in remodelling the ECM) and exosomes. Moreover, the importance of the bidirectional communication between cells and the ECM, as well as of ECM remodelling, has been increasingly acknowledged, as recently reviewed in detail by Werb and co-workers [17], amongst others [18,19]. The ECM functions not just as physical structural support but also regulates local concentrations of soluble factors and cell–cell interactions, in addition to ECM–cell direct interactions [18,19]. ECM is also a guiding scaffold for chemotaxis and tumour cell invasion [20]. ECM can regulate important cellular processes such as proliferation and migration, through activation of different signalling pathways (e.g., ERK and AKT) [21]. Tumour ECM composition is usually characterized by increased deposition of collagens, especially fibrillar types, as well as fibronectin and tenascin [22,23,24]. Moreover, ECM fibres present an aligned orientation that facilitates cell migration [25,26]. Enzymes related to ECM remodelling play a major role in cancer development, such as the MMP and lysyl oxidases (LOX). MMP are proteolytic enzymes that mediate matrix degradation, facilitate migration and invasion, promote angiogenesis and release ECM trapped growth factors [27,28,29]. LOX enzymes are responsible for collagen crosslinking, increasing matrix stiffness and are associated with enhanced tumour growth and progression [30]. Increased ECM stiffness is typically linked to tumour aggressiveness [31]. The recognition of the importance of the ECM in regulating developmental and oncogenic processes prompted research on biomaterials that can mimic the properties and dynamics of the ECM and development of scaffold-based 3D models [32,33,34,35].

In this review, we address the strategies developed to model the TME, with an emphasis on in vitro 3D co-culture approaches and their relevance for oncology research and anticancer drug discovery. We describe 3D TME models, including scaffold-embedded models, depicting each of the main non-malignant cell components, as well as co-culture strategies in which different TME cell components have been combined. Emphasis is put on the major findings in addressing the molecular crosstalk with tumour cells and effect on drug response. Advantages and caveats of 3D TME models will be discussed, as well as current needs and future perspectives.

## 2. Three-Dimensional Cancer Models 

Most cancer research and testing of drugs targeting the TME has been performed in syngeneic and xenograft mouse models, as reviewed extensively [36,37,38]. Although the majority of oncology research has been performed with monocultures of tumour cell lines, co-culturing of non-malignant TME cell types with tumour cells has been proposed and considered critical for depiction and evaluation of the interplay between tumour and TME components [39,40], increasing the clinical translation potential of the findings [41]. Co-culture approaches in 2D are widely used to study the crosstalk between tumour cells and TME components, either through direct cell–cell interactions or through paracrine signalling, making use of culture well inserts for compartmentalization of cellular components [42,43]. 

Although 2D methods are the most extensively used in the field due to their simplicity, three-dimensionality increases the level of recapitulation of the tumour tissue [44,45]. Differences in 2D and three-dimensional (3D) cultures have been demonstrated; specifically, 3D culture cell–cell interactions, cell–ECM interactions, and, consequently, cell polarity, gene expression and signalling pathways affecting proliferation, amongst other characteristics, present a greater resemblance to tumour cells in vivo [46,47,48,49]. Different 3D culture strategies, namely spheroids and matrix-embedded cultures, including organoids, have been extensively explored. 

### 2.1. Spheroids 

Multicellular tumour spheroids are spherical self-assembled aggregates of cancer cells that constitute a relevant and versatile tool [50,51,52,53,54]. Spheroids can be integrated with other platforms, such as embedding in scaffolds or culture in microfluidic systems [45,51,55], and their production can be easily scaled out and scaled up [56,57]. Spheroids can mimic tumour features observed in vivo, such as cell–cell and cell–ECM interactions and physicochemical gradients, with gene expression patterns closer to the original tumours than 2D cultures [50]. Particularly, spheroids with hypoxic and necrotic areas recapitulated more closely in vivo tumour gene expression profiles and exhibited the highest resistance to chemotherapy when compared to smaller and normoxic spheroids [58]. The main limitations regarding spheroids are related to the simplified architecture and ECM; autologous ECM is built up along culture time, limited to the components produced by the cell types constituting the spheroid [59,60]. Multiple methods can be used to obtain cell spheroids, namely gravity-based systems, in low adherence surface systems or agitation-based systems, such as spinner vessels and shake flasks; these methods have been extensively reviewed in recent years and a detailed description can be found, e.g., in the work of Rodrigues et al., 2020,and Costa et al., 2016 [45,50].

### 2.2. Tumour Organoids and Other Scaffold-Based Models

Patient-derived organoids have become an important tool in cancer research. These are 3D epithelial structures established from tumour tissues that self-organize and proliferate embedded in a matrix [61]. Importantly, organoids have been shown to sustain tumour cell heterogeneity and genetic properties of the original tumours over a series of passages [61,62,63,64]. This ability of propagation of epithelial malignant cells in vitro allowed for the establishment of biobanks for different types of cancer from multiple patients [65,66]. Moreover, organoids from metastatic gastrointestinal cancers were used in a co-clinical setting to assess drug response [67]. However, organoid technology still faces several challenges, from heterogeneous efficiency in derivation of organoids from distinct tumour types and individual patients [45] to difficulties in integrating vasculature, stromal and immune cells [45]. Nonetheless, preliminary successful co-cultures have been reported very recently [68,69]. 

One of the major limitations of organoids is the use of a reconstituted basement membrane extract (BME) secreted by a mouse sarcoma, commonly termed Matrigel [70]. Matrigel is a highly complex mixture, rich in type IV collagen, laminin, heparan sulphate proteoglycans and growth factors [70,71]. Matrigel or other brands of BME have been widely used to model the tumour matrix in vitro and in vivo but the animal origin, batch to batch variability, non-defined composition and presence of growth factors limit model reproducibility and introduce confounding factors [70,72]. 

Among the natural scaffolds, collagen I and fibrin have also been extensively used in cancer research [73]. An ideal scaffold should provide adequate environmental cues for cellular processes and ECM interactions while having a defined composition and being reproducible [45]. 

Artificial scaffolds, namely poly(ethylene) glycol(PEG)-based hydrogels and synthetic alternatives to Matrigel, are being extensively explored, as they allow for customized control of scaffold properties [74,75,76] but require deep knowledge on the interactions defining the TME that is still pending [77]. 

### 2.3. Microfluidic-Based 3D Models 

Microfluidics involve the use of microchips usually designed with a different number of chambers and lateral channels, with a fluidic flow [78]. These microdevices allow high spatial controllability but also require highly specialized skills and are usually low throughput and can only support short-term culture [79]. Fluidic shear stress needs to be finely tuned as high shear stress is reported to affect cell viability [80]. Recently, 3D bioprinting has been gaining momentum, as it allows for controlled cell distribution and can contribute to generate more complex models with higher reproducibility [81]. Nonetheless, in this case, the scaffold (bioink) choice is a crucial step [45,82]. 

## 3. Three-Dimensional Double Co-Cultures Incorporating Non-Malignant Cell Components of the Tumour Microenvironment 

### 3.1. Fibroblasts

**Figure 1:**
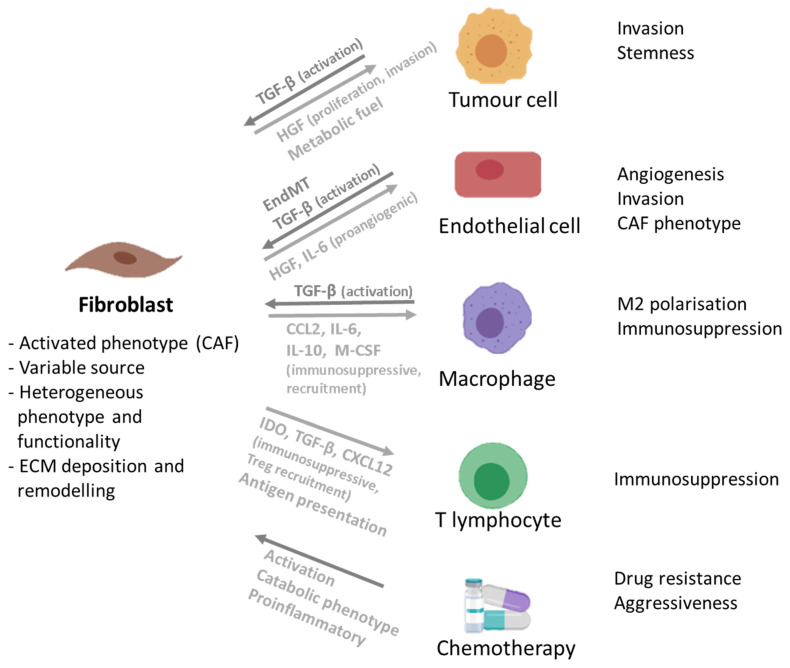
Schematic representation of cancer-associated fibroblasts’ (CAF) phenotype, their interactions and reciprocal effects on tumour cells and other non-malignant cells of the tumour microenvironment, and effects of chemotherapy [11,19,86,88,93]. Image created with BioRender. CAF: Cancer-associated fibroblast; CCL2: C-C motif chemokine ligand 2; CXCL12: C-X-C motif chemokine ligand 12; ECM: Extracellular matrix; HGF: Hepatocyte growth factor; IDO: Indoleamine 2,3-dioxygenase; IL-6: Interleukin 6; IL-10: Interleukin 10; M-CSF: Macrophage-colony stimulating factor; TGF-β: Transforming growth factor β.

In the tumour milieu, fibroblasts acquire an activated phenotype described as cancer-associated fibroblasts (CAF), a heterogeneous cell population, that represent the main stromal component of solid tumours [83,84]. This activated phenotype is mediated through multiple factors within the TME, mainly transforming growth factor β (TGF-β), but also fibroblast growth factor 2 (FGF-2) and platelet-derived growth factor (PDGF), paracrine factors secreted mostly by tumour cells [83]. Despite the lack of a consensus CAF molecular signature [84], α-smooth muscle actin (α-SMA) is described as a hallmark of the transition from fibroblasts to activated fibroblasts [85]. CAF produce bioactive molecules, such as ECM proteins, cytokines and growth factors, which influence tumour progression, invasion and drug resistance to different anticancer compounds [11,86,87,88] (Figure 1). In fact, CAF are one of the major producers of ECM and ECM remodelling mediators [19].

In recent years, CAF-targeting therapeutic agents have been proposed. These drugs can act by targeting CAF-derived factors (IL-6 or C-X-C motif chemokine ligand 12, CXCL12, inhibitors), or reverting their activated phenotype through TGF-β blocking, or directly targeting CAF subsets, such as fibroblast-activation protein (FAP) positive populations [89,90,91,92].

Several authors have described the isolation of fibroblasts from tumours followed by in vitro culture. It is safe to say that CAF are the non-malignant cellular type for which more protocols for isolation and culture are available. Multiple studies with fibroblast co-cultures are reported in the literature, although the majority employed fibroblast cell lines (Table 1). Regarding 3D co-cultures, fibroblasts are also the TME cell component more often incorporated, typically employing cell embedding, in a variety of biomaterials. 

#### 3.1.1. Models with Normal Fibroblasts

Aiming to mimic tumour–stroma cell interactions in initial stages of tumorigenesis, many authors resourced to normal fibroblasts. Kim et al. have proposed a proximity co-culture model, using a transwell system for co-culture of spheroids of a human colorectal cancer cell line (HT-29) and a normal colon fibroblast cell line on a collagen gel [94]. The authors reported activation of fibroblast towards a CAF phenotype in 3D but not in 2D co-cultures, with increased expression of α-SMA. Interestingly, a reciprocal interaction between the two cell types was demonstrated, with higher migration of the tumour cells in 3D co-cultures, which has been associated with epithelial to mesenchymal transition (EMT) mediated by TGF-β1 [94]. The same team later designed a microfluidic chip in which spheroids of HT-29 cells and normal colon fibroblasts were embedded in type I collagen, placed in separate channels at a microscale distance [85]. In this setting, fibroblasts also acquired phenotypic traits of CAF. Reciprocally, in co-cultures, tumour cells revealed increased migration, decreased proliferation and consequently reduced susceptibility to paclitaxel relatively to monocultures, compatible with EMT [85]. 

Froeling et al. used co-cultures of pancreatic cancer cell lines and non-tumorigenic stromal components: immortalized pancreatic stellate cells (isolated from normal pancreas tissue and considered the tissue resident fibroblasts) or a normal lung fibroblast cell line (MRC-5) [113]. Tumour spheroids were cultured over stromal cells plated on gels of collagen and Matrigel [113]. The authors have not addressed the phenotype of fibroblasts upon co-culture but observed phenotypic changes in tumour cells in co-cultures with both types of fibroblasts, such as the expression of molecules related to adhesion (E-cadherin, β-catenin and Ezrin), but not altered proliferation. Inert biomaterials have also been proposed to set up co-cultures of tumour cells and fibroblasts. Dondajewska et al. used co-cultures of a murine breast cancer cell line and a murine fibroblastic cell line in a natural inert scaffold made of silk fibroin [102]. Regarding the fibroblast phenotype, gene expression analysis suggested a transition to a CAF-like phenotype. They observed lower proliferation rates of tumour cells in 3D vs. 2D and co-cultures vs. monocultures. Moreover, monocultures displayed higher resistance to doxorubicin in 3D as opposed to in 2D, whereas differences between mono- and co-cultures were less pronounced. 

Cell encapsulation in inert hydrogels has also been explored for the generation of co-cultures of tumour cells and fibroblasts. Fang et al. used a microfluidic device with prostate cancer cells and normal prostate stromal myofibroblast cells (WPMY-1) co-cultured in two separate layers of alginate microcapsules to study paracrine interactions between the two cell populations [126]. As a proof-of-concept, the authors evaluated the shedding of E-cadherin byMMP, known to be deregulated in several cancers, including prostate cancer, and they found increased levels of shedding in co-cultures compared to monocultures [126]. In another approach, our team employed alginate microcapsules to co-culture a breast cancer cell line (MCF-7) with human dermal fibroblasts [101]. Deposition of ECM components, such as collagen I, as well as of secreted soluble factors, was detected in the microcapsules after two weeks of culture, compatible with transition to a CAF-like phenotype [127]. Moreover, tumour cells showed increased migration and angiogenic potential when in co-cultures [101]. This strategy was applied to lung cancer cell lines [56] and the feasibility of including other TME cell components has also been demonstrated with monocytic cells [128], which will be covered in Section 4.1.2. 

The effects of in vitro-activated fibroblasts on promoting proliferation of tumour cells and resistance to chemotherapy remains to be clarified, as contrasting results have been reported in different 3D co-culture setups, and the underlying mechanisms have not been elucidated. The model design criteria, e.g., source of fibroblast and tumour cell lines, ECM composition, distance between cells, interspersion of tumour cells and fibroblasts, and time of co-culture, will impact the local distribution of tumour- and fibroblast-derived soluble factors and ECM, modulating fibroblast activation and their reciprocal effect on tumour cells.

#### 3.1.2. Models with CAF and Non-Malignant Epithelial Cells 

Several studies have explored the effect of CAF in tumorigenic events, employing normal epithelial or benign tumour cells. Holliday et al., in 2009, developed a model of the breast cancer microenvironment that included luminal, myoepithelial cells and fibroblasts isolated from malignant or normal tissue, embedded in collagen I gels [129]. Once CAF were added to the aggregates formed by luminal and myoepithelial cells, a change in cellular organization occurred, with disruption of the basement membrane, leading to a change in the model architecture; this phenomenon was not observed with fibroblasts derived from normal tissue [129]. This disruptive effect was mostly abrogated with inhibitors of MMP or hepatocyte growth factor (HGF) receptor (c-MET), in accordance with other studies reporting the contribution of CAF-derived mediators to tumour progression [130,131]. Using Matrigel-embedded co-cultures, Shekhar et al., in 2001, also observed that CAF isolated from breast tumour tissue can stimulate the growth of non-tumorigenic and preneoplastic breast epithelial cell lines, while fibroblasts isolated from normal breast tissue induced the opposite effect [132]. 

Overall, these studies point to the ability of CAF to disrupt the normal epithelial cell architecture and phenotype, independently of the hydrogel in which cells were embedded, possibly linked to enhanced ECM deposition and altered ECM composition from CAF. Clark et al., in 2013, isolated CAF and normal fibroblasts from patient tissue and cultured them in 2D for two weeks in the presence of ascorbic acid to stimulate the secretion of ECM, prior to seeding on top a cell line representing a human benign prostatic hyperplasia (BPH-1) [120]. The authors observed changes in morphology and migration of the BPH-1 cells when in co-culture with CAF, compatible with a more invasive phenotype [120]. It would be interesting to characterize the composition of the secreted matrix and compare it to the ECM composition of the parental tumours from which they were isolated. 

#### 3.1.3. Models with CAF and Tumour Cells 

Jacobi et al. explored 3D co-cultures for evaluation of drug response in lung adenocarcinoma cell lines with distinct (epidermal growth factor receptor) EGFR profiles [98]. HCC827 cells, which harbour an EGFR mutation associated with clinical response to gefitinib, were co-cultured as spheroids with fibroblasts isolated from lung cancer tissues, within a scaffold of collagen I and Matrigel. The authors reported that HCC827 cells presented a higher invasive phenotype in the 3D co-culture than in monoculture. Moreover, once exposed to gefitinib (EGFR inhibitor) HCC827 exhibited higher drug sensitivity in heterotypic spheroids than in homotypic spheroids, whereas in 2D HCC827 cells were resistant to the drug. Increased drug sensitivity in 3D was associated with reduced signalling activity of Her family members, which might be due to increased gene expression and activation of members of the tumour necrosis factor-alpha (TNF-α) pathway in 3D. 

Other authors have also reported increased sensitivity to inhibitors of Her family members in tumour cell spheroids relatively to 2D cultures [133,134,135]. Nonetheless, the data are in opposition to findings by Wang et al. and Choe et al. [99,136], which reported increased anti-EGFR resistance in lung epithelial cancer cells co-cultured in 2D with CAF, mediated by stromal-derived HGF. Curiously, in double co-cultures of EC and tumour cells, no changes in drug sensitivity/resistance were observed, possibly due to the absence of a source of HGF [99]. It would be interesting to assess the production of HGF by fibroblasts within the 3D co-culture format proposed by Jacobi et al. and to understand how the presence of the ECM may affect the concentration of HGF in the extracellular milieu and the proximity of the tumour cells, given the role of ECM in regulating HGF/c-MET signalling [137].

Dolznig et al. generated multicellular tumour spheroids from colon adenocarcinoma cell lines and co-cultured them with CAF isolated from colon adenocarcinoma samples or normal fibroblast cell lines, all within a collagen gel stabilized with a nylon mesh [95]. The authors observed differences in cell–cell junctions and after four days of co-culture with CAF or normal fibroblasts and an invasive tumour cell phenotype, not found in tumour homotypic spheroids, even after 11 days of culture [95]. They reported a significant upregulation of gene sets involved in hypoxia, ECM deposition, EMT and angiogenesis in co-culture, in agreement with data obtained from patient samples [138]. On the other hand, Liu et al. observed prominent tumour cell invasive behaviour in co-cultures of a salivary gland adenoid cystic carcinoma cell line (ACC-M) with CAF isolated from an adenoid cystic tumour but not with a normal fibroblast cell line (HFL-1) [121]. In this study, the authors built a microfluidic device in which both cell types were embedded in BME (Cultrex) and patterned in two separate but connected chambers [121]. Moreover, this CAF-induced invasion was blocked by a MMP inhibitor [121]. 

In accordance with this, Attieh et al. also found differences between CAF and normal fibroblasts in inducing a tumour cell invasive phenotype, and this was linked to the collagen remodelling capability and fibronectin deposition by CAF. The authors generated tumour spheroids of mouse intestinal cancer cells (CT26) and embedded them in collagen I, together with CAF or their normal counterparts (both isolated from patients’ samples) [96]. Horie et al. also reported an increased potential of CAF relatively to normal fibroblasts to enhance invasion of tumour cells, in a very similar set-up of co-culture embedded in collagen [97]. A lung cancer cell line (A549) was co-cultured with CAF or normal fibroblasts isolated from tumour and healthy lung tissue, respectively. 

#### 3.1.4. Models with Different CAF Subsets 

The invasive prone co-culture phenotypes depending (or not) on fibroblast type emphasize the need for further studies on the heterogeneous nature of fibroblasts and their activation states. CAF heterogeneity has been identified in patient samples from different cancer types, namely, breast, pancreatic, gastric or colorectal cancers [93,139]. 

Su et al. identified different CAF subsets in patient samples, and linked one of them to chemoresistance [93]. Particularly, CAF were isolated from chemo-sensitive or chemo-resistant tumours and co-cultured in a transwell setting along with breast or lung cancer cell lines. The authors reported alleviation of growth inhibition of tumour cells when co-cultured with CAF from chemo-resistant tumours compared to CAF from chemo-sensitive ones [93]. In vivo, these CAF from chemo-resistant samples were identified as a specific subset expressing CD10 and GPR77 and secreting IL-6 and IL-8, which correlated with cancer stem cell enrichment. When IL-6 and IL-8 secretion was abrogated, the chemoresistance effect induced by CAF to cisplatin or docetaxel was not observed. Importantly, this CAF subset was correlated with worse prognosis in patient samples and was not distinguishable from other CAF in the expression of conventional markers, such as α-SMA, FAP or collagen I [93]. 

Herrera et al. found heterogeneous populations of CAF derived from primary colon cancer patients (*n* = 15). The authors classified CAF based on their ability to promote migration of a colorectal cancer cell line in a 3D setting and established a CAF promigratory signature, but the mechanisms underlying CAF heterogeneity have not been addressed. Nevertheless, this CAF signature was validated in Gene Expression Omnibus (GEO) database (accession number GSE51257, NCBI) and related to categorization of tumour progression risk in patients [140]. 

CAF heterogeneity is not only reflected in their phenotype but also function, as demonstrated with the identification of immunosuppressive, ECM secreting or antigen-presenting CAF, among others [141]. Another cause of CAF heterogeneity relies on their origin, as CAF can be recruited locally (tissue-resident cells), from adipose tissue or bone marrow. It has also been reported that CAF can be originated through epithelial or endothelial to mesenchymal transition [142,143]. A deeper understanding of the CAF-activation mechanisms is needed and has been attempted through the use of proteomics and single-cell RNA sequencing, aiming at identifying not only therapeutic targets but also putative prognostic biomarkers [141,144,145,146]. Importantly, in 2020 a consensus statement on CAF definition and biology has been issued. This was an effort to tackle the challenges of CAF heterogeneity and therapeutic targeting, and reinforcing the need for assay standardization [83]. In fact, CAF plasticity can augment modelling complexity and the impact of the culture format on tumour cell and CAF behaviour becomes clear—direct or indirect co-culture, proximity between the two cell types, tumour cells organized as 3D spheroids or as single cells, biomaterial composition, etc. It is therefore of paramount importance that the models employed for biological interrogation or drug assays are well-defined and properly characterized, including the phenotype of the fibroblast component. 

In summary, in vitro models based on 3D co-culture of tumour cells and CAF lead, in general, to a more invasive tumour cell phenotype, signs of EMT and higher drug resistance, which are features that can resemble the tumour progression in vivo. As mentioned earlier, proliferation levels are still a matter of debate, as contradictory observations have been found. All these studies highlight the important role of co-cultures in the discovery of CAF-secreted factors, such as ECM and soluble growth factors, and their role within the TME. Although most of the studies on drug challenge of tumour cell and fibroblasts co-cultures employed standard-of-care chemotherapeutics, co-cultures can also help clarify the therapeutic potential of CAF-targeting agents. 

### 3.2. Endothelial Cells

**Figure 2:**
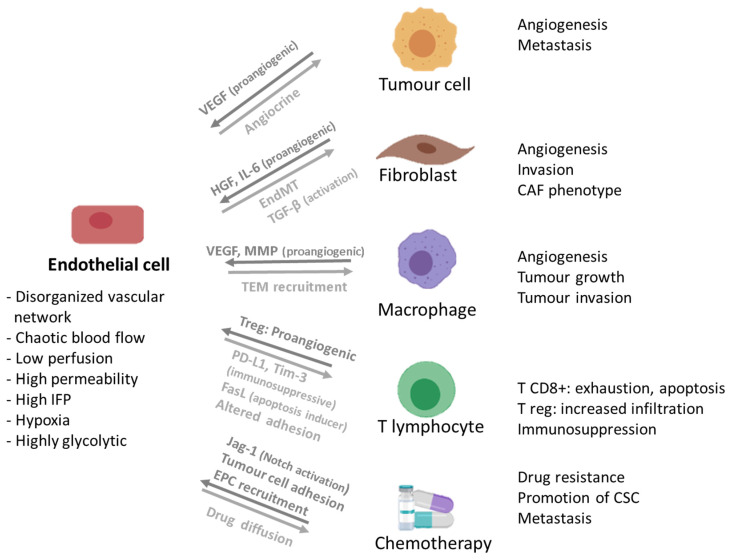
Schematic representation of the characteristics of tumour-associated endothelial cells, their interactions and reciprocal effects on tumour cells and on other non-malignant of the tumour microenvironment, and effects of chemotherapy on them [16,148,150]. Image created with BioRender. CAF: Cancer-associated fibroblast; CSC: Cancer-stem cell; FasL: Fas ligand; EPC: Endothelial progenitor cell; EndMT: Endothelial to mesenchymal transition; HGF: Hepatocyte growth factor; IFP: Interstitial fluid pressure; IL-6: Interleukin 6; Jag-1: Jagged-1; MMP: Matrix metalloproteinase; PD-L1: Programmed death ligand 1; TEM: Tie expressing monocytes; Tim-3: T-cell immunoglobulin and mucin domain-3; TGF-β: Transforming growth factor β; Treg: Regulatory T cell; VEGF: Vascular endothelial growth factor.

Angiogenesis is a recognized hallmark of cancer progression [16,147] and EC have been implicated in drug resistance processes [148] (Figure 2). Tumour blood vessels present several characteristics that differ from the regular ones, such as excessive branching, increased permeability and lack of pericyte coverage [16,149]. Moreover, tumour-associated EC are distinct from regular EC in terms of morphology, gene expression and metabolism, with tumour-associated EC exhibiting increased proliferation or loosen intercellular junctions [16,149,150]. These features augment the complexity of in vitro tumour modelling. 

#### 3.2.1. Heterotypic Spheroid Approaches

Heterotypic tumour spheroids are a versatile and simple 3D co-culture approach to incorporate EC [151]. Upreti et al., in 2011, generated heterotypic spheroids of a murine mammary tumour cell line combined with a murine EC line to assess the response to ionizing radiation or chemotherapy [152]. Firstly, the spheroids were generated by plating the tumour cells and, on day three, the EC were added. The EC were reported to infiltrate the spheroids towards the core, resulting in larger and more compact 3D structures than the tumour cell monocultures, which started to disintegrate after eight days. The presence of EC sensitized the tumour cells to chemotherapy. On the other hand, upon exposure to radiation, tumour cells in heterotypic spheroids had higher proliferation rates than in monotypic spheroids, suggesting a resistance mechanism that was not studied further. 

Chiew et al., in 2017, also employed a heterotypic spheroid approach, with tumour cell lines from hepatic and breast carcinomas and EC [153]. The authors observed the formation of tube-like structures of EC in co-cultures with HepG2, but not with the breast cancer cells lines MCF-7 and MDA-MB-231. The mechanisms underlying this difference remain to be addressed. The tube-like structures deteriorated after three days; still, the authors were able to set up drug challenge assays using tyrosine kinase inhibitors for 2 days and observed reductions in tumour cell viability and EC tube-like structures [153]. Shoval et al., in 2017, used not only tumour cell lines but also patient-derived tumour cells, together with human umbilical vein endothelial cells (HUVEC) [154]. The authors assessed essentially morphological aspects and the formation of capillary-like structures within the heterotypic spheroids. EC network formation was dependent on the tumour cell line used. It would be interesting to explore the pathways involved in this cell line-dependent behaviour and challenge the model with antiangiogenic drugs to consolidate its potential. 

Another combination of the 2D/3D approach was developed by Chaddad et al., in 2017, to co-culture spheroids from an osteosarcoma cell line with a monolayer of EC [155]. After 14 days of co-culture, the authors observed EC migration towards the tumour spheroid and formation of tubule-like structures, which was linked to higher levels of Vascular endothelial growth factor (VEGF) secreted by tumour cells at that time point in comparison to earlier culture days; there was also higher ECM deposition [155]. This system can provide clues to the factors important to perform successful co-cultures, namely VEGF concentration and ECM components, although, as observed by others, the tumour cell line can also impact EC tube-like formation efficiency. 

#### 3.2.2. Matrix-Embedding Approaches

A large proportion of models recapitulating steps of the tumour angiogenesis described in the literature explore cell or spheroid embedding in hydrogels. The use of hydrogels as ECM surrogates have been explored to define the spatial organization of the different cell types within the model and to mimic sprouting.

Ingthorsson et al., in 2010, co-cultured EC isolated from normal breast tissue with primary breast luminal and epithelial cells or normal and malignant breast cell lines, embedded in BME [156]. Two distinct cell compartments were seeded in different regions of the gel, without a physical separation. Increased proliferation and cloning efficiency of normal breast epithelial and tumour cells were observed in co-cultures. Control experiments in transwells suggested that these effects of EC over breast cells were at least partially associated with soluble factors derived from the EC, although their identification was not pursued. Tumour cell proliferation was higher in the vicinity of EC, which can be linked to higher concentrations of EC-derived soluble factors, due to diffusional gradients within the gel [156]. 

Chwalek et al., in 2014, proposed the use of glycosaminoglycan-based hydrogels (starPEG-heparin hydrogels) for co-culture of EC and tumour cells [77]. The authors explored the effect of biomaterial-associated parameters on the ability of EC to form capillary-like networks and tumour cells to proliferate, namely stiffness; content of RGD, a conserved tripeptide sequence recognized by integrins and other cell surface proteins; and growth factor content. Spheroids of the hepatic tumour cell line HepG2 were seeded into a hydrogel in which EC had previously formed tubular structures. The authors observed migration of tumour cells towards the EC and, reciprocally, infiltration of EC in the tumour spheroid [77]. Moreover, to increase EC culture duration, the authors combined EC with EC support cells, such as MSC, smooth muscle cells (SMC) and fibroblasts, and were able to maintain the tube-like network for 28 days. It would have been interesting to include tumour cells in these complex co-cultures. 

Roudsari et al., in 2016, explored bilayer PEG-based hydrogels to generate a layer model to study the impact of soluble vs. direct cancer cell interactions; a lung tumour cell line (344SQ) was cultured in one of the layers and HUVEC and EC support cells (human vascular pericytes) in the other layer [157]. EC tube-like structures were observed to be in contact with the tip of tumour cell projections and larger tumour cell clusters were formed in the proximity of the EC layer, which the authors suggested as being related to TGF-β secretion.

#### 3.2.3. Microfluidic Approaches 

In vitro systems of perfused human capillary networks have been extensively explored [158] and there have been attempts to integrate tumour cells into these systems to mimic tumour angiogenesis and provide models of vascularized tumours. 

Aref et al. employed a two-chamber microfluidic system to combine 2D and 3D culture approaches. Spheroids of the A549 lung adenocarcinoma cell line were cultured within a collagen gel in one of the chambers and the adjacent compartment contained a monolayer of HUVEC [159]. The authors reported signs of EMT, such as loss of E-cadherin and spheroid dispersion only in the co-cultures [159]. Furthermore, a challenge with drugs blocking EMT-related factors, such as EGFR inhibitors, reverted the EMT indicators [159]. These results were reported for co-cultures with the A549 cell line and not further validated with other tumour cells. 

Buchanan et al. employed a microfluidic approach with breast cancer cells (MDA-MB-231) surrounding a cylindrical central channel, in which EC in collagen formed a confluent layer with increasing shear stress [160]. Upregulation of proangiogenic genes in flow conditions compared to static has been reported, making this a suitable model to study the influence of hydrodynamic forces on tumour angiogenesis. 

In conclusion, co-culture of tumour cells with EC has been reported to depict features observed in vivo, particularly, signs of EMT or drug resistance (Table 2). Models employed usually portray specific events, such as tumour cell extravasation. Additionally, most models rely on HUVEC as an EC source and lack representation of the tortuous and leaky characteristics of the tumour vasculature [161]. 

### 3.3. Immune Cells

The development of in vitro models of tumour–immune cell interactions has gained importance [168] (Table 3), particularly since the emergence of the next-generation immunotherapies, such as immune checkpoint inhibitors. Multiple immune cells can be recruited to the TME, and the dynamics of tumour–immune cell interactions are complex and constantly evolving during tumour progression [169]. Innate immunity cells, such as macrophages, neutrophils, natural killer (NK) cells or dendritic cells (DC), can be found within the TME, as well as adaptive immune cells, T and B lymphocytes [170]. Tumour cells can escape immune surveillance through different mechanisms, such as immune checkpoint expression, and harbour an immunosuppressive environment [169,171]. Tumours can be classified as cold or hot, depending on their lack or abundance of immune cell infiltration, respectively. Cold tumours are characterized by an immunosuppressive TME, rich in immunosuppressive cytokines, such as IL-10 and TGF-β and presence of M2-like macrophages and T-regulatory cells (Treg); hot tumours are rich in T CD8+ lymphocytes, which have been linked to the ability to respond to immunomodulatory therapies [172,173].

#### 3.3.1. T Lymphocytes

Since the beginning of the last decade, there have been reports on differences in immunogenicity of tumour cells cultured in 2D or 3D. Dangles-Marie et al. observed that when moving from 2D tumour cell monolayers to spheroids, autologous cytotoxic T lymphocytes (CTL) exhibited less activation, as measured by their cytokine secretion, specifically interferon (IFN)-γ and TNF-α [185]. This evident decrease was not linked to a diminished major histocompatibility complex (MHC) I or tumour antigen expression, but due to a downregulation of Hsp-70, a protein required for cytoplasmic transport of processed peptides for MHC I presentation. It was hypothesized that this downregulation was due to the slower growth rate of tumour cells observed in 3D [185]. Ghosh et al. also found a differential activation of T cells when a metastatic melanoma cell line was cultured as a monolayer or spheroids [186]. In this study, the authors reported a decreased activation of CTL by specific melanoma-associated antigens in 3D, measured by IFN-γ cytokine secretion, associated with a decrease in expression of MHC-I and specific antigens [186]. In another report from the same team [198], the authors confirmed the reduced activation of CTL by melanoma cells in spheroids and pointed out possible mechanisms in addition to MHC I and antigen expression downregulation in 3D: the architecture itself, with less exposed cell surface in 3D; increased production of lactic acid, which was previously linked to suppression of DC activation; and polarization towards M2-like macrophages, contributing to an immunosuppressive TME [183,199,200]. These studies emphasize the relevance of a 3D setting to scrutinize potential mechanisms of tumour immune escape, although multiple factors in the TME are reported to promote an immunosuppressive environment [199]. 

A T cell infiltration model was described by Alonso-Nocelo et al. [187]. The authors seeded a lung adenocarcinoma cell line (A549) and a T lymphocytic cell line (Jurkat E6.1) on a porous polystyrene scaffold to generate 3D co-cultures [187]. The authors found a distinctive secretome in co-cultures, revealing proteins involved in angiogenesis, EMT and inflammation processes [187]. Furthermore, the complement system pathway was only activated in 3D cultures [187]. This work provided a method to study tumour–T cell crosstalk, although using a leukaemia cell line as a model for T lymphocytes; the translational of the platform to peripheral T cells remains to be demonstrated. 

Doumba et al. co-cultured autologous peripheral blood mononuclear cells (PBMC) with hepatic cancer cells or hepatocytes, in monolayers, and focused their analysis on T CD8+ cells, the major antitumour effector cells [192]. Both types of hepatic cells showed increased MHC-II expression when in co-culture with PBMC, and this correlated positively with the activation status of CD8+ T lymphocytes [192]. The mechanisms underlying this effect were not explored and the contribution of other PBMC subpopulations was not further investigated.

Recently, strategies to study immunotherapies such as adoptive cell therapies have been reported. Pavesi et al. tested engineered virus-specific T cells, previously shown to target hepatocarcinoma cell lines that express Hepatitis B Virus (HBV) antigens from naturally integrated viral DNA [201]. The authors developed a microfluidics approach, in which the central channel was populated with single cells or spheroids of a hepatic tumour cell line (HepG2) transduced to express HBV antigens, embedded in a collagen I gel. In parallel, the engineered T cells were added to one of the lateral channels. The authors observed T cell migration and induction of tumour cell death, whether using HepG2 as single cells or as spheroids. Furthermore, the authors tested T cell activity under hypoxia and observed reduced migration and a significant decreased antitumour effect in 3D, which were not observed in the 2D co-culture under hypoxia. This system can be applied to different cell types, as addressed in Section 4.1.1 [202]. Importantly, as in other studies, hypoxia and normoxia conditions need to be properly defined, namely the atmospheric oxygen vs. dissolved oxygen measurements [203]. 

Successful incorporation of intraepithelial lymphocytes in normal intestinal organoids of murine origin have been reported [204,205]. Briefly, the authors generated intestinal organoids in Matrigel and in parallel cultured intraepithelial lymphocytes isolated from the small intestine; after two days of monoculture, co-cultures were set up. The authors reported lymphocyte viability and motility for three or seven days, without or with cytokine (IL-2, IL-7, and IL-15) supplementation, respectively [204]. A similar work was performed in 2015 using murine enteroids and also intraepithelial lymphocytes [205]. Using patient-derived organoids, Dijkstra et al. used co-cultures of lung and colorectal cancer organoids with autologous T lymphocytes derived from peripheral blood [189]. Importantly, the authors found that T cells recognized tumour organoids but not organoids derived from healthy tissue. Thus far, studies with 3D co-cultures with Foxp3+ regulatory T lymphocytes (Tregs), which exhibit an immunosuppressor function, contributing to tumour growth, and are usually described as indicators of poor prognosis [206], have not been commonly reported. 

#### 3.3.2. Macrophages 

Tumour-associated macrophages (TAM) have a spectrum of phenotypes that can range from M1-like, conventionally described as pro-inflammatory and anti-tumourigenic, to the pro-tumourigenic and immunosuppressive M2-like, that typically populate tumours in advanced stages [207,208,209,210]. Nonetheless, TAM can exhibit a mixture of macrophage phenotypes in between these two prototypical states [210,211]. Recently, efforts to dissect these subpopulations, as well as other immune cell populations, exploring single cell analysis have been made [211]. A TAM signature expressing SLC40A1, which encodes for the iron exporter ferroportin, and GPNMB, which encodes the glycoprotein nonmetastatic melanoma protein B (NMB), was correlated with poor prognosis in hepatocellular carcinoma [212]. The iron transporter has been previously associated with an attenuation of macrophage-mediated immunity [213] and the glycoprotein NMB was found to be upregulated by TAM and induce cancer stem cell markers and tumour progression via binding to the CD44 in tumour cells, eliciting the expression of IL-33 and its receptor in different murine cancer models [214]. 

The use of human peripheral blood-derived monocytes is well established as a source of macrophages for in vitro assays and considered more relevant than the use of monocytic cell lines, such as RAW 264.7 and THP-1, which express lower levels of monocyte marker CD14 but constitute a virtual unlimited cell source [215,216,217]. Both peripheral blood-derived monocytes or THP-1 can be polarized towards macrophages, for example, respectively, by M-CSF and Phorbol-12-myristate-13-acetate (PMA) stimulation. Both cell types can be further polarized towards a M2 phenotype, through different factors (such as IL-10) or induced by the presence of tumour cells [175,218]. However, M2 polarized from THP-1 do not exhibit the same spectrum of gene expression and protein markers as monocyte-derived M2 [215,218]. Conversely, both primary macrophages and THP-1 cells can be polarized toward a pro-inflammatory M1 phenotype after exposure to inflammatory stimuli, such as lipopolysaccharide (LPS) and IFN-γ [215].

Regarding cell models depicting tumour–macrophage interactions, Maolake et al. employed a transwell approach to culture human prostate cancer cell lines and THP-1 [174]. They observed THP-1 polarization towards a M2-like phenotype and increased migration of tumour cells in the co-culture setting. The authors proposed the C−C Motif Chemokine Receptor 4 (CCR4) as a potential drug target; the receptor has been previously implicated in Treg recruitment and resistance to immunotherapy [219]. Tevis et al. generated heterotypic spheroids of a breast tumour cell line and a murine monocytic cell line, embedded in collagen [175]. They have also reported polarization into M2-like macrophages by detection of secreted IL-10. Drug challenge experiments showed less sensitivity of heterotypic spheroids to paclitaxel compared to tumour homotypic spheroids.

Noel et al., in 2017, combined human intestinal organoids seeded in a monolayer with macrophages derived from peripheral monocytes and studied cell–cell interactions [220]. This study was not performed with tumour cells but opens new avenues to pivotal research on recapitulating TME applying organoid technology.

#### 3.3.3. Neutrophils

As observed for other TME elements, neutrophils can also exhibit a dual phenotype, with anti- or pro-tumour action [221,222,223]. High numbers of tumour-associated neutrophils have been correlated with poor prognosis in patient samples [224]. However, the role of neutrophils is complex due to their plasticity and heterogeneity. In early stage lung cancer samples, a neutrophil phenotype compatible with antitumour functions due to induction of T cell proliferation and IFN-γ release was reported [222,225]. In murine models, neutrophils’ pro- or antitumour roles have also been described. Neutrophils favoured metastatic seeding of circulating tumour cells in a melanoma model, while decreased metastatic seeding in a breast cancer model [226,227]. Moreover, in murine models it was shown that neutrophil recruitment is dependent on granulocyte colony stimulating factor (G-CSF) and it can lead to a decreased tumour burden [221]. However, G-CSF-mediated neutrophil recruitment has also been linked to metastasis promotion [228]. Recently, it has been reported that the neutrophil effect was dependent on NK status [229]. This has been shown in murine models of breast tumour and in 2D triple cultures of breast cancer with neutrophils and NK cells, all from murine sources [229]. Particularly, neutrophils showed antitumour activity in the absence of NK cells. On the contrary, in the presence of NK cells, neutrophils played prometastatic roles by suppressing NK’s tumoricidal role.

Few in vitro models of the TME comprising neutrophils have been reported so far. There are reports describing 2D co-cultures of tumour cells and neutrophils, in which the latter also showed a pro- or antitumour effect [229,230]. As for 3D approaches, a report described high viability of a neutrophil-like cell line in scaffold of BME and collagen I; a microfluidic device has also been proposed to study neutrophil migration [231,232]. Still, both studies comprised only monocultures. A 3D co-culture of a carcinoma cell line and neutrophils has been proposed as a model of bacterial infection; still, their applicability in cancer modelling still needs to be explored [233].

#### 3.3.4. Dendritic Cells 

DC are antigen-presenting cells involved in T-cell-mediated antitumour immunity [234,235]. Depending on the tumour type, immature or mature DC can be found in the TME. Immature phenotypes express low levels of co-stimulatory molecules (CD80, MHC II) and secretion of low levels of immunostimulatory cytokines (IL-12), and therefore are unable to activate T effector cells, contributing to an immunosuppressive TME [183,236]. DC can be isolated directly from PBMC or differentiated ex vivo from monocytes; DC are then pulsed with tumour-derived antigens ex vivo and reinfused in patients, aiming at eliciting a T-cell response against endogenous tumour antigens (DC antitumour vaccines). Various DC-based clinical trials are ongoing and Sipuleucel-T, for prostate cancer, is the first FDA-approved cancer vaccine [237]. Nonetheless, there are several bottlenecks on DC-based vaccines, namely the definition of the most suitable maturation stimuli or the need for uniformization of manufacturing processes [238,239,240]. 

Regarding in vitro recapitulation of tumour–DC interactions, Gottfried et al. used co-cultures of spheroids from different cancer cell lines with human monocytes from peripheral blood that were differentiated into DC along the co-culture time with specific cytokines, widely used to differentiate DC from blood monocytes, namely IL-4 and granulocyte–macrophage colony-stimulating factor (GM-CSF) [183]. The authors reported cancer cell line-dependent modulation of DC, with different cytokine secretion profiles differentially modulating the DC phenotype. Moreover, lactic acid secreted by tumour cells and previously implicated in immunosuppressive M2-macrophage polarization impairs DC antigen presentation ability [183]. Using a microfluidics approach, Parlato et al. showed DC migration towards tumour cells driven by the CXCR4/CCL12 axis [184] and antigen uptake. A 3D model has been reported to study the interaction between DC and T lymphocytes, measuring maturation and contact duration. The authors reported enhanced interaction time with more mature DC; it would be interesting to include tumour cells in this setting [241].

#### 3.3.5. NK Cells 

NK cells are considered a part of antitumour immunity and are involved in cancer immunosurveillance [242]. Like DC, NK cells have been proposed for immunotherapy [243,244,245]. NK infiltration levels in tumours are described as low [206], though in patient samples from different cancer types in which NK numbers were highly detected, it was associated with a better prognosis [246,247]. In contrast, in peripheral blood, high NK detection was linked with poor prognosis for gastric cancer [248] but with an increased overall survival in colorectal cancer patients [249]. Methods to study 3D NK infiltration into tumour spheroids have been proposed, through stimulation with different cytokines, such as IL-2 and IL-18 [250]. Ayuso et al. developed a microfluidic system using a colon adenocarcinoma cell line (HCT-116) within a collagen gel seeded in a central microchamber and flanked by two lateral microchannels [178]. In one of these lateral microchannels, the authors added activated NK cells, isolated from PBMC. The authors observed increased migration of NK towards the tumour ones, even though tumour cell cytotoxicity induced by NK was not determined. 

#### 3.3.6. Other Immune Cells 

Regarding γδ T cells, a distinct subset of T lymphocytes with innate- and adaptive-like properties, pleiotropic roles of anticancer and pro-tumour have been described in mouse models, though elucidation on human γδ T lymphocytes is still pending [251,252]. A co-culture between spheroids of a lymphoma cell line and a subset of primary γδ T cells (TCRVγ9) derived from healthy donors was employed to study antibody-dependent cell cytotoxicity (ADCC) and response to Programmed cell death protein 1 (PD-1) blockade [253]. The authors found that γδ T infiltration in tumour spheroids was facilitated by anti-CD20 and detected ADCC evidenced by spheroid volume decrease. Moreover, this cytotoxicity was potentiated by an anti-PD-1. Importantly, the authors found similar results in an in vivo lymphoma model and detected the presence of this subset of γδ T lymphocytes in patient samples. 

Myeloid-derived suppressor cells (MDSC) constitute a heterogeneous immature population of myeloid cells with immunosuppressive functions [254]. In vivo studies have shown that MDSC can strongly suppress T cells and inhibit cytotoxic activity from NK cells [255,256]. MDSC levels have been proposed as indicators of poor prognosis, with detection of increased circulating MDSC numbers associated with advanced breast cancer [257]. In a recent study, cancer patient-derived MDSC were shown to induce a more pronounced inhibition of T cell proliferation than MDSC derived from healthy donors, in a transwell co-culture set-up [257].

### 3.4. Mesenchymal Stromal Cells 

MSC can be actively recruited by tumours and contribute to tumour promotion through diverse mechanisms [258]. Moreover, MSC are known for their plasticity and can differentiate into CAF, adipocytes or even EC and are associated with the build-up of an immunosuppressive TME [259]. Zhu et al. used stereolithography-based 3D bioprinting to fabricate a bone-like matrix with breast tumour cells (MDA-MB-231) co-cultured with bone marrow-MSC (BM-MSC) to mimic a bone metastasis environment [260,261]. In the presence of MSC (or osteoblasts), tumour cell growth was enhanced and VEGF secretion was increased in comparison to monocultures. Bersini et al. also used MSC differentiated into osteoblasts to study MDA-MB-231 extravasation to a bone-mimicking environment, in a microfluidic device [262]. Tumour cell extravasation through EC coated channels and migration were higher in the bone-like microenvironment than the collagen matrix [262].

Mosaad et al. used a co-culture of prostate cancer cell lines with BM-MSC, adipocytes or osteoblasts in a microwell platform. Tumour migration and proliferation were dependent on the specific tumour cell line aggressiveness, culture dimensionality and presence of stromal cells, with adipocytes leading to the highest tumour cell proliferation increase [263]. Moreover, drug assays in 3D co-cultures presented higher drug resistance to docetaxel but not to antiandrogen drugs [263]. The mechanism underlying these differences still needs to be clarified. Liu et al., in 2016, used a different source of MSC, from umbilical cord (UC-MSC) instead of bone marrow-derived ones, and set up a co-culture with a hepatocellular carcinoma cell line (HCCLM3) in alginate [264]. The authors observed enhanced tumour cell invasion capacity and increased expression of MMP genes and EMT-related genes (vimentin), but no differences in tumour cell growth profile or drug response to cisplatin between co-cultures and monocultures. In a 2D study by Chao et al., in 2012, UC-MSC co-cultured with a breast tumour cell line exhibited an antitumour effect [265]. Further understanding on MSC effects and their different sources is still required, especially as MSC have been proposed as a therapeutic vehicle for antitumour treatments [266,267]. 

Although the focus of this review is on solid tumours, TME recapitulation of non-solid tumours is also critical, as reviewed in [268]. Studies in 2D, with bone marrow accessory/stromal cells to mimic events of haematological malignancies, are common in the literature [269,270]. Purroy et al. aimed to mimic the chronic lymphocytic leukaemia (CLL) microenvironment in bone marrow [270]. The researchers used co-cultures of CLL cells isolated from patient’s PBMC and bone marrow stromal cells (human cell line UE6E7T-2) [270]. In this co-culture system, tumour cells showed a proliferative phenotype comparable to the one found in vivo and found chemoresistance linked to upregulation of antiapoptotic proteins. Moreover, 3D co-cultures to depict the bone marrow microenvironment in different diseases have also been attempted [271,272,273]. A 3D approach using inert scaffold in which MSC derived from patient samples and differentiated into osteoblasts were co-cultured with myeloid leukaemia cell lines [274]. The authors found increased ECM deposition, though it was not identified its composition, and cell cycle arrest in the 3D vs. 2D culture setting. Upon chemotherapy challenge, fewer apoptotic cells were observed in 3D co-cultures than in monoculture and 2D. When combined with an integrin blocking agent, drug sensitivity was restored in 3D co-cultures, possibly due to disruption of cell adhesion and migration.

### 3.5. Other Non-Malignant Cell Types of the TME 

Additional non-malignant cell types, such as cancer-associated adipocytes and SMC, can also be present in the tumour milieu [9]. Regarding adipocytes, there is evidence that cancer-associated adipocytes can secrete adipokines involved in tumour growth and immune evasion, dedifferentiate into fibroblasts and provide free fatty acids for tumour cell consumption [275]. A 2D co-culture of adipocytes and monocytes (THP-1 cell line) in transwell revealed that THP-1 were induced to differentiate towards a M2 phenotype in presence of adipocytes [276]. Yue et al. developed a co-culture of tumour cells and adipocytes. The authors embedded the 3T3 mouse fibroblast cell line in microwells loaded with a hydrogel mixture of PEG and methacrylated gelatine, and induced differentiation of 3T3 to the adipocytic lineage [277]. After seven days, the authors introduced triple negative breast cancer cell lines, which formed spheroids inside the microwells, surrounded by adipocytes. The presence of tumour cell lines increased matrix stiffness relatively to co-culture with non-malignant murine mammary spheroids. Furthermore, when using two hydrogels with different stiffnesses, adipogenesis was inhibited with higher stiffness when in the presence of tumour cells. As adipocytes are described to secrete cytokines involved in promoting angiogenesis [278], it would be interesting to incorporate EC in this system, as matrix stiffness also affects angiogenesis [279]. In fact, using a different 3D approach, Agarwal et al. incorporated EC with adipocytes and a breast tumour cell line (MCF-7) in a microfluidic device and observed increased stiffness as well [280]. Additionally, EC formed tube-like structures and drug resistance was increased in triple cultures than in double co-cultures without EC or in 2D monolayers. Herroon et al. used co-cultures of adipocytes (murine bone marrow derived) and prostate tumour cells (PC3) and found increased tumour spheroid volume in co-cultures [281]. In a parallel setting, using single cells in layered matrices, the authors observed increased invasion of tumour cells into the adipocyte layer. Unfortunately, the authors did not identify the paracrine factors involved in this crosstalk.

Devarasetty et al. used colon carcinoma cell lines and SMC isolated from rabbit colonic submucosa [282]. The authors embedded tumour spheroids and SMC in collagen I gels and observed that tumour cells exhibited decreased proliferation and less migratory potential (in terms of cell projections and EMT markers) in the SMC-containing matrix than in the collagen I alone. This phenotype was related to the differences in the matrix topography, with higher fibre alignment and organization due to ECM remodelling by SMC. In fact, there is evidence that aligned fibres can facilitate migration of tumour and other stromal cells [283]. Moreover, the authors challenged the cultures with 5-FU and observed that drug sus-ceptibility was dependent on the tumour cell line, but still lower susceptibility of the tumour cells within the SMC-matrix than in collagen only was observed. This could be linked with tumour cells diminished proliferation levels detected in the SMC-matrix. It is not clear if SMC are preserved along time or only their derived matrix is retained.

## 4. Combination of Multiple Non-Malignant Cell Types 

**Figure 3:**
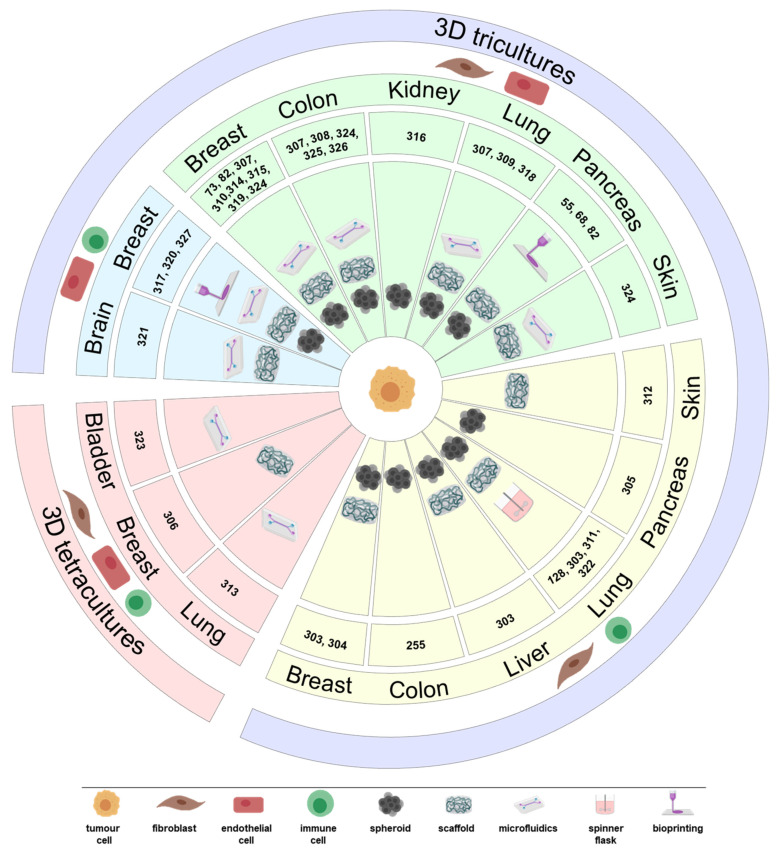
Schematic diagram showing examples of co-cultures of tumour cells with two or more tumour microenvironment cell components (fibroblasts, endothelial or immune cells) using different 3D approaches heterotypic spheroids and scaffold-based methods (artificial or animal-derived), employing distinct culture systems, such as spinner-flasks and microfluidic devices, and innovative techniques for generation of the 3D cultures, such as bioprinting [55,68,73,82,128,195,303,304,305,306,307,308,309,310,311,312,313,314,315,316,317,318,319,320,321,322,323,324,325,326,327]. Created with Biorender icons.

The multiple cell types present in the TME can have several effects on tumour progression, metastasis and therapeutic outcome. Several reviews have focused on the role of these multiple cell types [84,284,285,286]. In addition to the crosstalk between tumour cells and other cell types, the interplay between different non-malignant cell types within the TME is also important. As examples, fibroblasts can either lead to T cell suppression by secreting different factors, e.g., TGF-β and CXCL12, or increased effectiveness of T cells by enhancing their recruitment, highlighting CAF phenotypic and functional heterogeneity, although a T CD8+ immunosuppressive function seems to be more prevalent among CAF functions [287,288,289]; EC can also produce factors that hamper T cell function [290]; on the other hand, EC can decrease the expression levels of adhesion molecules, such as intercellular adhesion molecule 1 (ICAM-1), involved in leucocyte recruitment, contributing to a reduced immune tumour infiltration [291]; CAF-derived factors have been reported to promote angiogenesis [16,292,293] and act synergistically with EC to induce chemoresistance [294]; macrophages are reported to secrete VEGF and FGF-2, two known proangiogenic factors [295]. Antiangiogenic therapy, namely anti-VEGF, has been correlated with increased lymphocyte infiltration and immunotherapy effectiveness [296,297]. However, anti-VEGF therapy has also been associated with reprogramming of CAF to a more proangiogenic phenotype, in a VEGF-independent manner, through the secretion of PDGF-C, and polarization of macrophages towards a M2-like immunosuppressive phenotype [298]. TAM can physically exclude CD8+ T lymphocytes and prevent their antitumour function [299]. Drugs targeting macrophages, such as colony stimulating factor 1 receptor (CSF1R) inhibitors, have shown limited antitumour effects in patients [300] and a role of CAF in this resistance has been identified, for example, by recruiting pro-tumorigenic MDSC [301]. Combination therapy of CSF1R inhibitors with chemotherapeutics or other immune-modulating therapies is under clinical trials [302]. It is therefore crucial to map and understand the complex and multifactorial set of cellular interactions within the TME that contribute to cancer progression and therapeutic resistance. Experimental human-relevant models in which these multifactorial interactions can be interrogated, potential targets identified, and combinatorial therapies targeting multiple TME effectors tested. Development of cancer co-cultures with more than two cell types is a step closer to achieve those models (Figure 3) and in the next sections triple and tetracultures will be discussed. 

### 4.1. 3D Tricultures

#### 4.1.1. 3D Tricultures of Tumour and Stromal Cells

Various groups established culture models to recapitulate interactions between tumour, endothelial and stromal cells within the TME. Correa-Sampaio et al. developed a cell model to mimic the sprouting stages of tumour angiogenesis by generating heterotypic spheroids of a breast cancer cell line (MDA-MB-231), fibroblasts and EC (HUVEC), embedded in a collagen I gel [73]. The authors observed that EC formed networks intertwined with fibroblasts and that these sprouts (defined as HUVEC outgrowth from the spheroids) were in higher numbers in the presence of tumour cells than in double cultures of EC and fibroblasts. The authors challenged the model with antiangiogenics, such as anti-VEGF, and observed a reduction in the endothelial network, exhibiting less branching. 

Ehsan et al. also combined tumour, fibroblasts and ECs but used a two-step approach [307]. First, lung, breast or colon tumour cell lines were combined with human EC (umbilical cord-derived) to generate heterotypic spheroids; then, spheroids were embedded on a fibrin gel with normal human lung fibroblasts [307]. The spheroids exhibited signs of sprouting angiogenesis and tumour cells migrated to the surrounding matrix, especially under hypoxic conditions [307].

The inclusion of fibroblasts has already been reported to promote the formation of capillaries [328]. Amann et al. developed a heterotypic spheroid-based triculture of EC (HUVEC and Human primary microvascular EC, HMVEC), a fibroblast cell line and two non-small cell lung cancer lines [329]. The authors used two approaches: direct co-culture of the three cell types or combining tumour cells and fibroblasts and adding the EC after 5 days. At day 10, no EC were detected in the direct triple cultures, but in the sequential approach, EC migrated towards the central area of the spheroid, where fibroblasts were located and hypoxic cores formed. Two antiangiogenic drugs were tested, with no statistically significant differences in EC migration. We have recently reported a strategy for long-term culture of triple heterotypic spheroids of EC (HUVEC), human dermal fibroblasts and a breast cancer cell line (HCC1954), under agitation; in accordance with other authors, we observed that EC were localized in close vicinity to fibroblasts, in the core of the spheroids [310]. In our system, EC maintenance for up a month of culture was dependent on the tumour cell line, the presence of fibroblasts, which secreted collagens I and IV, and agitation but occurred even without formation of hypoxic cores. 

Tricultures with BM-MSC as source of stromal cells have also been proposed. Lamichhane et al. generated heterotypic spheroids of human BM-MSC combined with a lung adenocarcinoma cell line (A549) and human pulmonary microvascular EC (HPMEC) [330]. At day 15 of culture, only 0.1% of the initial seeded EC were present, though MSC were maintained [330]. The authors reported higher reactive oxygen species (ROS) levels in 3D than in 2D tricultures, which may have a pro- or antitumorigenic role [331]. In drug response assays with chemotherapeutics (gemcitabine and paclitaxel), no significant differences in cell death were observed between 3D triple cultures and 2D tricultures, despite the upregulation of ABC-B1, efflux transporter associated with drug resistance [330]. These findings require further clarification and suggest that although this might be a feasible platform for the three-cell type combination, improvements regarding EC viability and functionality in the presence of MSC are required. Bray et al. also performed triple cultures of tumour, MSC and EC, using acute myeloid leukaemia cells (cell lines or primary), all embedded in starPEG-heparin hydrogels; EC survival and formation of EC-tube-like structures was favoured in this system [332]. Upon chemotherapy challenge, increased drug resistance was detected in the triple cultures vs. 3D and 2D monocultures. A CXCR4 antagonist led to decreased tumour-EC contacts in 2 out of 3 primary samples but did not affect viability in the three samples. Work from the same group used the same hydrogel for setting up triple cultures with breast and prostate cancer cell lines. They described similar results in terms of EC behaviour and chemotherapy resistance [333]. Moreover, the same authors showed the versatility of this system by applying it to triple cultures with primary fibroblasts (instead of MSC), EC–HUVEC or HMVEC, and the non-malignant MCF10A cell line. Still, the triple cultures of EC, MSC and breast tumour epithelial cells were not performed. 

Applying a microfluidic approach, Rogers et al. used co-cultures of MDA-MB-231, fibroblasts (human or murine) and EC (HMVEC) [334]. The researchers seeded fibroblasts within a collagen I and Matrigel scaffold in the central chamber of the device and one tumour spheroid was added on top. EC were seeded in one of the lateral channels. Fibroblasts formed fibril-like structures and tumour cells preferentially migrated along these fibrils. Lee et al. also used a microfluidic device for the generation of tricultures of lung tumour cells (A549), murine fibroblasts (3T3) and EC (HUVEC) [309]. Cells were seeded sequentially: the tumour cells embedded in collagen I were placed in a microwell, fibroblasts in collagen I were seeded on top of the tumour cells, and EC were then seeded in a channel placed on top of the microwell. Tumour cells and fibroblasts spontaneously aggregated into a heterotypic spheroid and HUVECs formed vessel-like structures along the channel, and some reached and interacted with cancer cell spheroids. In the absence of fibroblasts, these tube-like structures were not observed, reinforcing the concept that fibroblasts sustain EC. When exposed to chemotherapeutic agents (paclitaxel and gemcitabine), tumour cells from cultures containing fibroblasts presented higher drug resistance [309]. In double co-cultures of fibroblasts and tumour cells, increased expression of genes involved in pathways related to metastasis and angiogenesis were observed over time. Although Rogers et al. and Lee et al. performed triple cultures, their analyses focused on tumour-fibroblast interactions; it would be relevant to characterize the EC population.

Pape et al. used scaffolds of collagen and laminin to set up triple cultures of colorectal cancer cell lines, HUVEC and normal fibroblasts or CAF (patient-derived) [308]. The authors found that in the presence of CAF, there was an induction of a tumour invasive phenotype with increased expression of HGF and Tissue inhibitor of metalloproteinases 1 (TIMP-1) and disruption of the EC network, not observed with normal fibroblasts. Herrera-Perez et al. developed a co-culture model with glioblastoma-derived cell lines and astrocytes and/or endothelial precursors, embedded in a matrix of collagen and hyaluronan [335]. The researchers observed that, in the presence of astrocytes, all glioblastoma cell lines tested exhibited higher migration, while in double or triple cultures with endothelial precursors, different migration effects were observed, depending on the cell line [335]. Endothelial precursors impaired migration in a stem-like population of glioblastoma cells. This finding might seem controversial as vascular networks can constitute migration routes but, in this study, endothelial precursors did not form tube-like structures. 

Altogether, these data point out the relevance of stromal–endothelial interactions to support EC in heterotypic cultures along with tumour cells but also that these processes are largely influenced by the tumour cell line and origin of the stromal cells. Further research will be required to pinpoint the critical molecular components required to sustain EC in these complex models. The EC-tube-like phenotype seems to be favoured in 3D triple cultures of with tumour cells and fibroblast relatively to double co-cultures with tumour cells. This constitutes a step closer to recapitulating in vivo processes, still improvements are needed regarding culture duration. 

#### 4.1.2. Triple Cultures with Immune Cells

Concerning studies including immune cell populations, several models have been proposed incorporating monocytic cells. Linde et al. set up co-cultures of skin squamous cell carcinoma cell lines, human dermal fibroblasts and macrophages differentiated from PBMC-derived monocytes. Fibroblasts and monocytes were embedded in collagen gel and tumour cells were placed on top [312]. The study was performed with human cells and murine equivalents in parallel [312]. The authors showed M2 macrophage polarization after three weeks of co-culture, both when using human or murine tumour cells. Moreover, they observed increased tumour cell protrusions within the hydrogel in the presence of both fibroblasts and macrophages or only macrophages, but not with fibroblasts only. This was linked to increased detection of MMP in the presence of macrophages [312]. Similar results were reported by Liu et al. on co-cultures of tumour cell lines (lung adenocarcinoma) with fibroblast and monocytic cell lines, all embedded in a collagen gel [311]. In triple cultures, the authors found the highest expressions of MMP-1 and VEGF, two factors known to be involved in tumour progression, invasion and angiogenesis [336,337]. 

Our group explored a strategy based on an inert biomaterial, alginate, to generate triple co-cultures of tumour cells, fibroblasts and monocytes and evaluate cellular crosstalk without the interference of active exogenous ECM and soluble factors [128]. For this, spheroids of non-small cell lung cancer cells (NCI-H157 cell line), human fibroblasts and human monocytes (PBMC-derived or THP-1) were co-encapsulated in alginate [128]. This triple culture (3D-3 culture) exhibited characteristics of an immunosuppressive TME, such as secretion of immunosuppressive cytokines (IL-4), and matrix remodelling enzymes (MMP-1) and accumulation of ECM components. Moreover, monocytes infiltrated the tumour spheroids, polarizing towards a M2-like phenotype without supplementation of growth factors and cytokines. Drug challenge with cisplatin and a CSF1R inhibitor (BLZ945) induced modulation of genes associated with macrophage transition from M2 to M1, as previously reported [338,339]. This culture system has been recently expanded to breast cancer cell lines [340]. 

Aiming at recapitulating the complexity of the immune infiltrate, several authors developed co-cultures with whole PBMC. Hoffmann et al. used two models, heterotypic spheroids of tumour cell lines, CAF and PBMC and explants derived from tumour samples [195]. Both models showed that were amenable to drug challenges with different chemotherapeutic compounds (e.g., 5-FU and SN38, the active metabolite of irinotecan) and targeted agents, such as cetuximab (anti-EGFR) and trastuzumab (anti-Her2) [195]. Due to easier availability, the spheroid model with cell lines is proposed for drug screening, while explants are proposed for precision medicine approaches, although it is not clear if the stromal compartment in patient-derived explants was preserved, since the authors only mentioned the lack of EC. Co-cultures of spheroids of a breast cancer cell line (MDA-MB-231) and PBMC derived from breast cancer patients have also been proposed as an approach to test immunotherapies [341]. Further elucidation of the immune cells infiltrating and their preservation in this culture setting is needed, as well as the relevance of employing just one tumour cell line. Koeck et al. generated heterotypic spheroids of lung cancer cell lines (Calu-2 and A549) and a fibroblast cell line (SV80), to which PBMC were added after 10 days [342]. The authors observed that, in the presence of fibroblasts and with the Calu-2 cell line, PBMC did not migrate, remaining in the spheroid periphery. Using A549, in double culture with PBMC, and in triple cultures, PBMC were visible in the central area of the spheroid. Moreover, the authors characterized immune cell types present and observed more activated CD8+ T lymphocytes in triple cultures than in double cultures. Phenotypic status and PBMC infiltration were dependent on the tumour cell line. For example, T lymphocyte infiltration was decreased in triple vs. double cultures when using Calu-2 cell line, but no differences were found with A549, while activated NK were increased in triple vs. double cultures when using the A549 cell line and no differences were found when using Calu-2. Nonetheless, triple cultures showed increased levels of cytokine secretion, including chemokines, independently of the tumour cell line used. Further dissection on these interactions would clarify the mechanisms underlying these observations, but it seems that the increased activation of T cells in the triple cultures is counterbalanced by possible physical limitation to infiltration due to the fibroblast presence; therefore, it would be interesting to analyse ECM components. Herter et al. also followed a similar approach but for colorectal cancer and tested immunotherapeutics: heterotypic spheroids of tumour and fibroblast cell lines were co-cultured with PBMC [343]. The authors challenged the system with a variant of IL-2, a bispecific antibody that binds to carcinoembryonic antigen (CEA, an antigen overexpressed on different tumour cells) and to CD3 (T lymphocytes), and the combination of these. The latter was more effective than monotherapy on T, NK and NKT cell activation and tumour cell death [343]. It would be interesting to test the system with patient-derived tumour cells and address infiltrated lymphocytes, which usually are suppressed and exhibit suboptimal activity [343]. 

A microfluidic approach was used to explore the effects of monocytes in T cell-based immunotherapies [202]. The authors have previously developed a microdevice with engineered T cells and tumour cells [188]; and here the authors generated tumour cell spheroids from a hepatic tumour cell line (HepG2) transduced to express HBV antigens and embedded the tumour aggregates with monocytes isolated from PBMC in a collagen I gel. The gel was injected in the central chamber of the microdevice, and T cells were added to only one of the lateral channels. A significant decrease in T cell antitumour activity was observed in the presence of monocytes, which was reversed once checkpoint inhibitors were used, namely anti-PD-1 or anti-programmed death-ligand-1 (PD-L1) antibodies. Importantly, this inhibitory action on T cells exerted by monocytes was not observed in a 2D setting. This study highlights the importance of a 3D context to study such interactions and highlight the clinical relevance of increased PD-L1+ monocytes identified in HBV-associated hepatocarcinoma patient samples [344]. Importantly, the suppression of T cell activity by monocytes was also dependent on the method of T cell engineering, making this a pertinent model to study different CAR-T cell manufacturing approaches and their interaction with the TME [202]. These 3D co-cultures incorporating different immune cells lack full characterization and profiling of the immune compartment. Still, they can constitute a useful tool to explore immunotherapeutics once better characterized. Triple cultures of immune cells and fibroblasts have a lot of room for improvement as CAF can exert an immunosuppressive effect and most 3D cultures fail to recapitulate this phenomenon or do not address it. 

Regarding triple cultures of tumour, immune and EC, only few studies are reported [317,320,321,327] and include mainly monocytes as the immune cell type; therefore, advances on these tricultures are needed as EC-immune interactions are involved in defining an immunosuppressive TME and influence therapeutic response [345,346].

### 4.2. Tetracultures

Co-culture strategies have been expanded to include an increased number of cell types and better recapitulate the network of cellular interactions within the TME. Xu et al. used a microfluidic chip to recapitulate lung cancer microenvironment using a lung cancer cell line (A549), a human bronchial epithelial cell line (16HBE), HUVEC, a human lung fibroblast cell line (WI38) and a mononuclear cell line (THP-1), all in different compartments [313]. They observed changes in fibroblast and monocytes towards pro-tumourigenic phenotypes. This was most probably due to the interaction with cancer cells. In a reciprocal manner, they found markers of EMT and invasion in tumour cells co-cultured in presence of the other cell types [313]. Tang et al. aimed to mimic glioblastoma TME by employing bioprinted scaffolds of gelatine methacrylate with hyaluronic acid, the main component of brain ECM, and co-cultured monocytes (differentiated from THP-1 or induced pluripotent stem cells, iPSC) with neural populations (astrocytes and neural stem cells) and glioblastoma cells derived from xenografts [347]. The authors found an enrichment in hypoxia response genes, stemness markers and genes associated with an invasiveness signature. Drug challenge with EGFR inhibitors and temozolomide showed enhanced resistance in tetracultures compared to tumour homotypic spheroids. Neufeld et al. also developed a bioprinted platform to mimic the glioblastoma microenvironment, but used a different bioink based on fibrin and gelatine [348]. The authors combined tumour cells from cell lines or patient-derived with primary astrocytes, microglia in a perfusion chip with channels covered with EC (HUVEC) and pericytes. This pentaculture system showed similar results with mouse models than the 2D in terms of tumour growth and transcriptional profiles, and drug response to a P-selectin inhibitor. Langer et al. used a bioprinting approach to design a versatile tetraculture system [82]. The authors used tumour cell lines or patient/xenograft-derived breast or pancreatic tumour cells, fibroblasts and EC, and adipocytes or MSC according to their relevance in the TME of specific cancer types. They observed ECM deposition and formation of EC-tube-like structures. Drug response to different drug modalities was assessed: more increased doxorubicin resistance was seen in 3D as opposed to 2D tetracultures and sunitinib (a receptor tyrosine kinase inhibitor) inhibited the EC network. 

Focusing on recapitulating specific features of the metastatic process, multiple studies have been developed to mimic cell types and ECM characteristic of the metastatic site [349]. Jeon et al. aimed to assess cancer cell extravasation by using a microfluidic device to harbour a bone-mimicking environment through co-culture of BM-MSC, osteoblast (differentiated from MSC) and EC (HUVEC) in a fibrin gel [350]. In this setting, EC formed branched structures and MSC colocalized and adhered to EC, suggesting a supportive role, like mural cells or pericytes in vivo. Afterwards, a bone-seeking clone of a breast metastatic cancer cell line was added. Tumour cell adhesion and extravasation towards the bone-like ECM was higher than towards the matrix without stromal cells. Interestingly, a control experiment was performed using a myoblast cell line instead of osteoblasts and the events described above were significantly decreased. Furthermore, the authors identified the A3 adenosine receptor as potentially responsible for the lower extravasation of cancer cells into the muscle-mimicking environment [350]. The same group, applying a similar experimental microfluidic approach, found that MDA-MB-231 extravasation was mediated by C-X-C Motif Chemokine Receptor 2 (CXCR2) in tumour cells and the chemokine CXCL5 secreted by the bone mimicking environment, as CXCR2 blocking decreased extravasation [262].

These multiple co-culture systems depict specific events according to the study goal. The inclusion of multiple cell types poses greater challenges as it increases modelling complexity, as addressed in the Section 5.

## 5. Concluding Remarks and Future Perspectives 

In summary, the studies performed with 3D heterotypic models were important to determine the molecular mechanisms governing the TME and recapitulate in vitro those molecular interactions. Despite the intensive TME model development in recent years and their enormous potential, the field faces various drawbacks and challenges. Several variables underlying 3D modelling, namely, the methodology to obtain the 3D architecture (spheroid, scaffold, bioprinting), the culture platform (agitation or static systems, microfluidics), model setting (direct or indirect cell contact and cell distance), cell source (cell lines or primary cells; human or animal-derived), scaffold source (human, animal or synthetic) and properties (bioactivity, stiffness), among other specifications. All of these features can impact the study outcome, cause confounding factors and therefore need to be carefully addressed. This variability hinders a direct comparison of the outcomes. Even with similar approaches, difficulties in interpreting results can emerge from the use of different readouts and timepoints of analysis. In fact, the plethora of in vitro 3D models of the TME have in common the need for increased comparability and reproducibility. 

### 5.1. Choice of Scaffold 

Scaffold-based approaches are numerous and scaffold selection can interfere with the contributions of the different cellular types. The choice of biomaterial to mimic ECM has been an extensively investigated subject [35,351,352,353]. Different matrices can impact the outcome of the experimental study [354]. The same tumour cell lines in Matrigel-embedded spheroids exhibited enhanced growth in comparison to spheroids embedded in collagen [42] or in chitosan-alginate [193]. Collagen gels are conventionally used as the tumour ECM is abundant on collagens and collagen I is typically associated with migration of tumour cells [355]. However, tumour-derived ECM are much more complex and ECM specificity has been reported, suggesting that matched tumour-derived matrices may improve the recapitulative power of 3D co-cultures. Along with collagen, Matrigel has also been broadly used in 3D models, but it has a murine origin and presents high batch-to-batch variability, which pose the evident problem of experimental reproducibility [356]. Therefore, other matrices with defined compositions have been proposed, such as other natural hydrogels such as alginate or artificial-based scaffolds [353,357]. Not only does biomaterial composition need to be taken into account, but its stiffness does as well, as this can impact cellular phenotype and tumours are described to present a higher stiffness than normal tissues [75,358,359,360]. Paszek et al. observed that mammary epithelial cells seeded in stiffer gels led to loss of organization and disrupted adherens junctions, while in softer gels they maintained mammary acini architecture [359]. In 2016, a study using a breast cancer cell line (MCF-7) and different percentages of alginate showed not only differences in cell morphology in the 2D vs. 3D approach, but also that the highest proliferation rate occurred in the softest hydrogel, resembling the initial stages of tumour formation [358].

Overall, biomaterial selection should consider adequate environmental cues for cellular processes and ECM interactions and has defined composition and is reproducible [45]. Artificial scaffolds, namely PEG-based hydrogels, allow for customized control of scaffold properties [74,75] but require a high level of knowledge of the interactions defining the TME, which is a challenge per se [77]. For organoids, engineered synthetic matrices functionalized with different motifs, such as collagen peptide GFOGER and laminin motif IKVAV, have been reported but did not show the same performance as Matrigel in terms of organoid formation efficiency [361]. 

The experimental setting based on physical contact between cells or distance, only allowing paracrine signalling, should be selected according to the study’s purpose. In this light, microfluidic approaches are becoming widely employed and allow this type of control. Still, they can restrict downstream analysis due to the small cell number and amount of mRNA available in the microdevice chambers [164]. Improvements on cell retrieval and single cell analysis have been remarkable [362,363]; still, they are very expensive and technical demanding.

### 5.2. Choice of Cell Source 

Concerning cell source, in several studies, murine tumour or TME cells are used. Moreover, the tumour type and the origin of the stromal components seldom do not match, which may introduce confounding factors to the model. Although the representativity of tumour cell lines has been debated, and a lot of cell line-dependent effects have been reported [364,365,366], they represent a virtually unlimited source in comparison to the scarce tumour patient material. Cell lines should be carefully selected, and a panel of cell lines should be employed for correct interpretation of the results and formulation of conclusions. 

In addition to these considerations on cancer cell lines, there is also the open question on the use of normal fibroblasts instead of CAF, as phenotypic and functional differences have been reported, though this should be dependent on the study’s purpose. Some studies aim to portray fibroblast activation by tumour cells; therefore, normal fibroblasts should be employed; when aiming to assess the reciprocal CAF effects on tumour cells, there is a need to carefully address the phenotype of the CAF-representing component, as the activation state may vary depending on source and culture conditions. The culture time is also an important parameter, as the amount of time required to induce CAF activation and/or observe CAF effects on tumour cells will vary, depending on the fibroblast origin (normal or CAF). In fact, the timeframe for inclusion of different cell types can also affect the outcome of the co-cultures. Sequential addition may favour previous build-up of favourable/detrimental microenvironments influencing certain cell types, as observed with sequential seeding of fibroblasts in tumour spheroids, resulting in more homogeneous distribution of fibroblasts and reduced formation of necrotic cores [108]. Sequential inclusion can also help define the spatial distribution [108,329], which can be enhanced using microfluidics and bioprinting techniques.

Patient-derived cells are being increasingly employed to develop precision medicine approaches [367]. Patient-derived tumour organoids are reported to be representative of tumour cell heterogeneity [65,66] and have been proposed as a preclinical tool for drug development [368]. However, organoids are strictly epithelial, and the addition of TME components has been hampered by difficulties in fine-tuning the culture conditions to allow carcinoma cell expansion and survival of TME non-malignant cells [369]. Recently, a few studies reported important breakthroughs in the co-culture of organoids with immune cells and inclusion of vascularization [69,370], which will be for sure further explored and refined in the near future. A few studies tried to tackle the limitations of propagation and viability of primary human cells by performing expansion in murine models, and then proceed to in vitro 3D cultures [87,371]; the evidence cumulated so far point to retention of patient heterogeneity along expansion; however, the limitation in terms of non-malignant cells from the patient tumour remain.

Ex vivo approaches, such as explants and tissue slices, represent the tumour heterogeneity, TME heterogeneity and architecture, and are amenable to drug challenges, including immunotherapeutics [372,373]. However, ex vivo approaches are limited by the amount of patient material and need technical improvements regarding culture viability and long-term preservation of TME elements ex vivo [374]. 

### 5.3. Physicochemical Parameters 

Hypoxia is prevalent in the TME and can influence the phenotype and behaviour of different cell types and even drug diffusion, as described in [375,376,377,378]. Therefore, the systems should be well characterized in terms of oxygen availability, by measuring dissolved oxygen and evaluate oxygen diffusion within the 3D structures to precisely define cellular normoxia or hypoxia. Culture medium requirements can also vary between cell types, which is also a current limitation for complex co-cultures. An example is the effect of lactate, typically accumulated in immunosuppressive TME, on immune cells activity and motility. In particular, DC and T lymphocytes are negatively impacted by lactate, which also induces macrophage polarization towards pro-tumoral M2-like phenotypes [183,255,379,380]. 

### 5.4. Current Challenges and Applications 

In conclusion, there are still significant challenges in generating recapitulative TME in vitro models, though they represent multiple advantages, not only in terms of time and cost but also in allowing one to define and control microenvironmental parameters and isolate the effects of each component. 

In order to simulate systemic interactions, multiple organ-on-a-chip devices based on microfluidics have been recently under intense development, with advances being made in bioprinting, as reviewed by Radhakrishnan et al. [381]. Despite their great potential, the recapitulation of each individual organ faces the bottlenecks mentioned for the 3D TME model; in particular, recapitulating immune system complexity is a major challenge [382].

Regarding drug challenge experiments, critical parameters are the duration of drug exposure, the drug concentrations and the readouts for assessment of drug effect, all profoundly influenced by the cell model characteristics [383,384]. Furthermore, the controls should be carefully interpreted, as several studies compare 3D co-cultures to the 2D setting and in distinct timepoints, but differences between 3D and 2D are largely acknowledged and the comparison with the real tumour/clinical setting is most often lacking. The challenges regarding the application of 3D cancer models to drug assays has been discussed in detail by Langhans et al., 2018, and Nii et al., 2020 [35,385].

We envisage that the 3D cancer modelling field, especially strategies based on co-culture to depict the TME, will be critical to dissect the full network of molecular interactions underlying cancer progression. Despite the advances, there is a plethora of open scientific questions that could be effectively investigated employing 3D TME models. 

CAF have been described to have a pro-tumorigenic role within the TME, but most of the studies were based on the analysis of the differential impact of normal fibroblast vs. CAF-like cells on the tumorigenicity of cancer cells. Still, the molecular mechanisms that drive normal fibroblast activation, from a healthy tissue context to a pro-tumorigenic CAF phenotype in tumorous tissues, are not well defined. This knowledge will open the possibility to counteract the CAF activation process within the TME. It is plausible to hypothesize that this is a long process, similarly to the majority of tumorigenic developmental processes. Two-dimensional approaches for co-culture of tumour cells and fibroblasts are normally short-term due to an intrinsic property of the 2D system - cell proliferation is limited by the available surface area, which may not be sufficient to induce the transition from normal fibroblasts to the CAF phenotype. Three-dimensional co-culture strategies enable substantially longer culture times compared to 2D ones and can therefore be helpful to shed light on this topic. Specifically, we envisage the use of long-term 3D co-cultures of normal fibroblasts present in the tissue of origin, matched with tumour-specific cell spheroids. Studies will take advantage of powerful analytical techniques such as mass cytometry and single-cell RNA sequencing analysis of the isolated fibroblasts to evaluate the potential molecular signals associated with CAF differentiation; the recently developed spatially resolved variations of both analytical techniques can reach a new level of knowledge on the influence of tissue architecture on intercellular communication. 

Immunotherapy approaches have been proposed for “cold” tumours, such as high-grade gliomas [386] and other solid tumours, but immunosuppression represents one of the hurdles against the effectiveness of these therapeutic modalities, as, for example, antitumour CAR-T technology have been successfully applied against leukaemia’s such as B cell acute lymphoblastic leukaemia, but CAR-T therapies are still not considered very effective against “cold” solid tumours [387]. This occurs mostly because of the immunosuppressive TME in solid tumours (e.g., secretion of immunosuppressive cytokines, presence of inhibitory immune-checkpoint ligands and immunosuppressive cells, namely Treg and M2 macrophages) and the lack of accessibility towards tumour cells due to the dense ECM and chaotic vascular network. There is robust evidence that TME reconstruction approaches based on 3D co-culture can depict the immunosuppressive milieu that is typical of those “cold” tumours; therefore, these strategies can play a critical role in studying and counteract the immunosuppressive signals. 

The 3D co-culture approaches may enable us to study CAR-T chemotaxis toward the tumour and especially CAR-T infiltration. ECM deposition reduces T-cell tumour penetration into lung tumour slices [388] and reduced T cell cytotoxicity against tumour cells has been reported after T-cell culture in the high-density collagen matrix [389]. In this regard, 3D cell culture models enabling native ECM deposition within 3D multicellular structures [128] provide suitable platforms to analyse and challenge such phenomena in a reconstructed 3D TME, with pre-clinical and clinical translation potential. Under this perspective, we foresee the application of representative advanced 3D tumour cell models, which enable the co-culture of tumour cells, stromal, immune and EC to assess the synergistic therapeutical potential of novel immunotherapies and antiangiogenics [346]. 

### 5.5. Towards Increased Comparability and Reproducibility of 3D TME Models 

Finally, quoting the famous sentence by the statistician George Box, “Essentially, all models are wrong, but some are useful”, it is important to point out that an ideal, universal 3D TME model is not envisioned. Still, they are collectively very useful in basic and applied cancer research and are expected to continue to occupy an important position, as enabling tools in tumour cell biology, oncoimmunology and anticancer drug discovery. As it was made clear in this review, many methodologies exist to set up 3D cell models in parallel with a plethora of readouts to interrogate these models, employed in a multitude of applications. It is important to stress that this abundance claims for a collaborative long-term effort of the transdisciplinary research community focused on 3D model development, characterization and validation. The collaborative effort should also be focused on assay readout development and standardization, with the objective to develop a toolbox of less time-consuming, user-friendly analytical tools, with spatial resolution for widespread application to distinct 3D model set-ups [390]. Moreover, establishment of 3D cell model validated guidelines and common methodologies is also critical for the definite acceptance of 3D TME models as human in vitro alternatives to understand how to challenge tumours cells and the TME, aiming at providing better therapeutic solutions for cancer patients.

## Figures and Tables

**Table 1:** Examples of studies developing and employing 3D co-cultures of tumour cells and fibroblasts.

Tumour Cells		Fibroblast Source	Platform and Matrix	Main Outcomes	Ref.
Cancer Type	Source				
Colorectal	Human cell line (HT-29)	Human cell line (CCD-18Co)	Collagen I Microfluidics	Increased spheroid size but decreased cell proliferation in co-culture; lower sensitivity to paclitaxel in co-culture	[85]
	Human cell line (HT-29)	Human cell line (CCD-18Co)	Transwell	Fibroblast activation and increased tumour cell migration and proliferation in co-culture; no resistance to 5-FU in co-culture	[94]
	Multiple human cell lines (HCT116, Caco-2)	Human primary (CAF and NF)	Collagen with nylon mesh	Increased signs of tumour cell invasion and enrichment in pathways involved in hypoxia, ECM, EMT and angiogenesis; no differences between CAF and NF	[95]
	Murine cell line (CT26)	Human primary (CAF and NF)	Collagen	Increased signs of tumour cell invasion in co-culture with CAF in comparison with co-culture with NF or tumour cell monoculture	[96]
Lung	Human cell line (A549)	Human primary (CAF and NF)	Collagen	Increased signs of tumour cell invasion and no differences in tumour cell proliferation in co-cultures with CAF	[97]
	Human cell lines (HCC827, NCI-H1975 and NCI-H1437)	Human primary (CAF)	Spheroids in collagen I and Matrigel	Increased signs of tumour cell invasion and decreased drug resistance to EGFR inhibitor in co-cultures	[98]
	Human cell lines (PC-9 and HCC827)	Murine cell line (MRC5)	Transwell	Increased drug resistance to EGFR inhibitor in co-cultures	[99]
	Human cell line	Murine cell line (WA-mFib)	Transwell Gelatine microspheres	CAF activation measured by αSMA Enhanced tumour cell invasion	[100]
Breast	Human cell line (MCF-7)	Human primary (dermal NF)	Spheroids in alginate microcapsules Spinner flasks	Loss of tumour epithelial phenotype, deposition of ECM proteins and increased proangiogenic potential in co-cultures	[101]
	Murine cell line (EMT6)	Murine cell line (NIH3T3)	Silk fibroin	Acquisition of CAF phenotype in co-culture, with decreased proliferation and signs of EMT; Enhanced resistance to doxorubicin in 3D vs. 2D monocultures than in co-cultures vs. monocultures	[102]
	Human cell lines (BT474, T47D, MCF-7 and SKBR3)	Human primary (CAF and NF)	Spheroids	Fibroblast infiltration dependent on tumour cell line	[103,104]
	Human cell lines (UACC-893, BT20, MDA-MB-453)	Human primary (foreskin NF)	Rotary suspension	Cancer cell invasion into fibroblast core; Deposition of ECM proteins	[105]
	Human cell lines (MDA-MB-231 and MCF-7)	Human primary (breast CAF and skin NF)	Spheroids	Increased tumour cell proliferation and migration in co-culture with CAF but not NF; Increased α-SMA in CAF co-cultured with MDA-MB-231; NF not activated by MDA-MB-231 or MCF-7	[106]
	Human cell lines (T47D, MDA-MB-361 and MDA-MB-231)	Human primary (dermal NF)	Spheroids	Similar tumour growth in mono and co-cultures; No differences between mono and co-cultures in sensitivity to combination of chemotherapy and radiotherapy	[107]
	Human cell line (MCF-7)	Murine cell line (MRC-5)	Spheroids	Increased tumour cell growth in co-cultures, with formation of necrotic spheroid cores.	[108]
	Human cell line (MCF-7)	Murine cell line (3T3)	PET scaffold Microbioreactor with agitation	Increased resistance to tamoxifen, oxaliplatin and cisplatin in co-cultures	[109]
	Human cell lines (MDA-MB-231 and MCF-7)	Human cell line (HTB-125)	Collagen I Microfluidics	Increased signs of tumour cell invasion, collagen deposition and stiffness in co-culture	[110]
	Murine primary mammary tumour cells	Murine primary (CAF)	Organoids Matrigel	Co-culture increased signs of invasion through release of TGF-β	[111]
	Human cell line (MDA-MB-231)	Human mammary fibroblasts (HMF)	Spheroids Alginate and Collagen I	Increased tumour and fibroblast invasion through alginate and collagen mixed gel than collagen only matrix Invasion potentiated by CXCL12-secreting fibroblasts	[112]
Pancreatic	Human cell line (Capan-1 and Paca-3)	Murine cell line (MRC-5); human immortalized	Collagen I and Matrigel	No alterations in tumour cell proliferation; Modulation of adhesion molecules	[113]
	Human cell line (Patu8902)	Human immortalized	Spheroids Bioprinting	Generation of heterotypic spheroids	[114]
	Human cell line (PT45)	Human primary (CAF or normal)	Microcarriers Spinner flask	ECM deposition in co-cultures with NF and CAF; NF acquired activated phenotype	[115]
	Human cell lines (PANC-1, AsPc-1, BxPC-3, Capan-1 and MIA PaCa-2)	Human primary (CAF)	Spheroids	Spheroids more compact in co-culture, with collagen deposition; Higher gemcitabine resistance in co-culture than tumour monospheroids	[116]
Lung Colorectal Esophageal Pancreatic	Patient-derived xenografts	Human primary (CAF)	BME	Drug resistance to different chemotherapeutics in co-culture	[87]
Lung, breast, pancreatic	Human cell lines (e.g., A549, MCF-7, Panc1)	Murine cell line (MRC5); Human primary (CAF) and cell lines	Spheroid	Increased proliferation in co-cultures; Differential secretion of cytokines depending on the tumour cell line; Decreased drug sensitivity to targeted therapy in co-culture for lung tumour cell lines, but not for breast cancer cell lines	[117]
Liver	Human cell line (HepG2)	Murine cell line (3T3-J2)	Spheroids Collagen	Higher drug resistance to doxorubicin in co-cultures	[118]
	Murine and human primary mammary and breast tumour cells	Murine and human primary (CAF)	Transwell Organoids Matrigel	Co-culture increased organoid growth but not organoid number In response to sorafenib, regorafenib or 5-FU, less organoid growth inhibition in co-cultures	[119]
Prostate	Human cell line (BPH-1)	Human primary (CAF and NF)	Fibroblast produced matrix	Increased signs of migration and invasion in co-culture	[120]
Salivary gland adenoid cystic carcinoma	Human cell line (ACC-M)	Human Primary and cell line (HFL1)	BME Microfluidics	Increased signs of invasive phenotype in co-cultures with CAF, but not with NF MMP inhibitor blocked CAF-induced invasion	[121]
Ovarian	Human cell line (OVCAR5)	Murine cell line (MRC-5)	Matrigel Bioprinting	Generation of co-cultures with different sizes and cell densities	[122]
Breast Pancreatic	Cell lines (murine 4T1 and human MDA-MB-231 and Panc-1)	Murine and human cell lines (3T3, BJ-hTERT) and human primary (CAF, NF)	Spheroids	Increased α-SMA and collagen in co-culture; Decreased penetration of nanoparticles in co-culture	[123]
Breast Lung	Human cell lines (MCF-7, SKBR3, A549)	Human primary (CAF from chemo-sensitive or chemo-resistant tumours)	Transwell	Increased drug resistance in co-cultures with CAF isolated from chemo-resistant tumours	[93]
Breast Lung	Human cell lines (T47D, MCF-7; H1299)	Murine cell line (MRC5) and human NF	Spheroids	Fibroblast localized preferentially in the inner part of the spheroids; identification of specific compounds that inhibited fibroblast migration	[124]
Breast Colorectal	Human cell lines (MCF-7 HCT-116)	Primary (human dermal NF)	Spheroids Microfluidics	Imaging and quantification of tumour cell spheroid invasion into fibroblast spheroid	[125]

BME: Basement membrane extract; CAF: Cancer-associated fibroblast; ECM: Extracellular matrix; EGFR: Epidermal growth factor receptor; EMT: Epithelial to mesenchymal transition; MMP: Matrix metalloproteinase; NF: normal fibroblasts; PET: Polyethylene; 5-FU: 5-Fluorouracil; α-SMA: alpha-smooth muscle actin.

**Table 2:** Examples of studies developing and employing 3D co-cultures of tumour and endothelial cells (EC).

Tumour Cells		EC Source	Platform and Matrix	Main Outcomes	Ref.
Cancer Type	Source				
Liver Breast	Human cell lines (HepG2, MCF-7 and MDA-MB-231)	HUVEC	Spheroids	EC formed tube-like structures in co-cultures with HepG2 but not with breast cancer cell lines; structures declined after 3 days of culture	[153]
Breast	Human cell lines (MCF7, T47-D and MDA-MB-231)	Primary (breast tissue)	BME	Increased breast cancer spheroid growth (size and proliferation) in co-culture with EC	[156]
	Murine cell line (4T1)	Murine tumour-like EC line (2H1)	Spheroids	EC infiltrated tumour spheroids; increased sensitivity to chemotherapy in co-culture but not to radiation	[152]
	Human cell line (MDA-MB-231)	Human dermal microvascular cells	Microfluidics	CXCR4 or CXCR7 on breast cancer cells promoted adhesion to EC	[162]
	Human cell line (MDA-MB-231)	HUVEC	Collagen and fibrin Microfluidics	Increased MDA-MB-231 invasion in collagen, in co-culture	[163]
	Human cell line (MDA-MB-231)	Immortalized microvascular EC	Collagen Microfluidics	EC formed a confluent layer aligned with flow direction; upregulation of proangiogenic genes in flow vs. static conditions	[160]
Lung	Human cell line (A549)	HUVEC	Spheroids Microfluidics	Evidence of EMT in co-cultures, reverted by EGFR inhibitor	[159]
Melanoma, Breast, Pancreatic	Multiple cell lines or patient-derived	HUVEC	Spheroids	Formation of capillary-like structures	[154]
Liver	Human cell line (HepG2)	HUVEC	Glycosaminoglycan-based hydrogel	EC infiltration and tumour cell migration in co-culture	[77]
Colorectal	Human cell line (HCT-116)	Human Colonic microvascular EC	Matrigel Microfluidics	EC formed tube-like structures; gemcitabine nanoparticles decreased tumour cell proliferation	[164]
Colorectal Glioma	Cell lines (rat glioma C6 and human colorectal LoVo and HT29)	HUVEC	Microfluidics Matrigel	Spheroid secreted higher levels of VEGF than monolayers; EC formed more tube-like structures in 3D than in 2D co-cultures.	[165]
Glioma/Glioblastoma	Patient-derived glioma cell line (GB3)	HUVEC	Matrigel and fibrin Microfluidics	Enhanced tumour cell migration in co-culture and no effect on proliferation; CXCR4 inhibitor decreased tumour cell migration.	[166]
	Human glioblastoma cell line U87MG	HUVEC	Spheroid Microfluidics Fibrin	EC tube-like formation towards tumour spheroid Antiangiogenics (bevacizumab and sunitinib) reduced EC migration	[167]

BME: Basement membrane extract; CXCR4: C-X-C chemokine receptor type 4; EC: Endothelial cells; EGFR: Epidermal growth factor receptor; EMT: Epithelial to mesenchymal transition; HUVEC: Human umbilical vein endothelial cells; VEGF: Vascular endothelial growth factor.

**Table 3:** Examples of studies developing and employing 3D co-cultures of tumour and immune cells.

Immune Cells		Tumour Cells		Platform and Matrix	Main Outcomes	Ref.
Cell Type	Source	Cancer Type	Source			
Monocytes/Macrophages	Human monocytic cell line (THP-1)	Prostate	Human cell line (BHP-1)	Transwell	Polarization to M2-like macrophages; increased tumour cell migration	[174]
	Murine leukaemia cell line (RAW 264.7)	Breast	Human cell line (MDA-MB-231)	Spheroids embedded in collagen	Polarization to M2-like macrophages; increased resistance of tumour cells to paclitaxel in co-cultures	[175]
	Murine BM-derived macrophages		Murine cell line (Py8119)	Spheroids embedded in Matrigel	Macrophage infiltration in 3D; Increased tumour cell invasion in co-culture	[176]
	Peripheral blood- derived	Pancreatic	Human cell line (Panc-1)	Microfluidics	Increased macrophage migration in co-culture or induced by flow Partial blocking of macrophage migration by anti-IL-8 and anti-CCL2	[177]
Natural killer cells (NK)	Peripheral blood- derived	Colorectal	Human cell line (HCT-116)	Collagen; Microfluidics	Increased migration of NK towards tumour cells	[178]
	Engineered NK cells (CAR-NK)	Colorectal	Human primary	Organoids in Matrigel	CAR-NK recognizing different antigens; increased cytotoxicity of CAR-NK towards tumour organoids than normal counterparts.	[179]
	Human cell line (NK-92)	Lung	Human cell line (A549)	Spheroids Transwell	Enhanced migration and cytotoxicity of NK in the presence of CXCL12	[180]
	Peripheral blood- derived	Cervical	Human cell lines (CaSki, SiHa)	Spheroids	NK infiltration in spheroids and cytotoxicity towards tumour cells	[181]
	Peripheral blood- derived	Liver	Human cell line (HepG2)	Spheroids	NK-mediated tumour lysis mainly at the periphery of the spheroids	[182]
Dendritic cells (DC)	Monocytes from peripheral blood	Urothelial Melanoma	Several human cell lines	Spheroids	Distinct DC phenotypes dependent on tumour cell line	[183]
	Monocytes from peripheral blood	Colorectal	Human cell line (SW620)	Microfluidics	DC migration towards tumour cells; histone deacetylase inhibitor and IFN-α led to increased DC migration, through activation of CXCR4/CCL12 axis	[184]
T lymphocytes	Human CTL clone	Lung	Human cell line (IGR-Heu)	Spheroids	Less CTL activation in 3D co-cultures than in 2D	[185]
	Human CTL clone	Metastatic melanoma	Human cell line (HBL)	Spheroids	Tumour associated antigen recognition by CTL decreased in 3D	[186]
	Human cell line (Jurkat E6.1)	Lung	Human cell line (A549)	Transwell	Co-culture secretome enriched in proangiogenic and proinflammatory EMT-inducing factors.	[187]
	Engineered T cells	Liver	Human cell line (HepG2)	Microfluidics	T cell infiltration and induction of tumour cell death	[188]
	Peripheral blood lymphocytes	Colorectal and lung	Human organoids	96-well U-bottom	Autologous tumour T cells recognized tumour organoids but not healthy counterparts	[189]
	αβ T cells carrying a transgenic TCR peptide-specific	Colorectal	Human organoids	BME	Engineered T cells induced death of antigen-specific tumour cells	[190]
	CAR-T	Lung Breast	Human cell line (A549; MDA-MB-231)	Microfluidics Porcine decellularized matrix	CAR-T decreased tumour cell volume and increased tumour apoptosis relative to untreated or co-culture with non-engineered T lymphocytes	[191]
PBMC	Human	Liver	Human primary hepatocytes (tumour and healthy)	2D	T CD8+ showed increased activation but less viability in co-cultures	[192]
	Human	Prostate	Cell lines (human LNCaP, C4-2, C4-2B and murine TRAMP-C2)	Spheroids in chitosan– alginate or Matrigel	Decreased proliferation of tumour cells in comparison to Matrigel; immune cells infiltrated the tumour spheroids	[193]
	Human	HNSCC	Human cell line (EpCAM-positive FaDu)	Spheroids Spinner flask	Immune cell infiltration into spheroids; bispecific antibody (anti-EpCAM and anti-CD3) alone or combined with cisplatin decreased spheroid viability	[194]
	Human	HNSCC	Human cell lines (UD-SCC 4, 5, 6)	Spheroids	Anti-EGFR antibody induced leukocyte infiltration into tumour spheroids and the effect was abrogated by anti-CCL2 antibody	[195]
	T and NK from healthy donors or patients	Colorectal	Human cell line (HT-29) or primary	Spheroids	T and NK infiltration into spheroids and increased tumour apoptosis in co-cultures; both processes enhanced by IL-15 supplementation	[196]
	Treg and NK	Breast	Human cell lines (MCF-7 and MDA-MB-231)	Matrigel	Model establishment and implementation of analytical methods (RNA extraction, immunohistochemistry)	[197]

BM: Bone marrow; BME: Basement membrane extract; CAR: Chimeric antigen receptor; CCL2: C-C Motif Chemokine Ligand 2; CTL: Cytotoxic T lymphocyte; CXCR4: C-X-C chemokine receptor type 4; CXCL12: C-X-C Motif Chemokine Ligand 12; DC: Dendritic cells; EGFR: Epidermal growth factor receptor; EMT: Epithelial to mesenchymal transition; HNSCC: Head and neck squamous cell carcinoma; IFN-α: Interferon alpha; IL-15: Interleukin 15; EpCAM: Epithelial cell adhesion molecule; NK: Natural killer cells; TCR: T-cell receptor; Treg: Regulatory T cells.

## Abbreviations

5-FU	5-Fluorouracil
ADCC	Antibody-dependent cell cytotoxicity
BM	Bone marrow
BME	Basement membrane extract
CAF	Cancer-associated fibroblasts
CAR	Chimeric antigen receptor
CEA	Carcinoembryonic antigen
CCL2	C-C motif chemokine ligand 2
CSF1R	Colony stimulating factor 1 receptor
CTL	Cytotoxic T lymphocytes
CXCL12	C-X-C motif chemokine ligand 12
CXCR4	C-X-C chemokine receptor type 4
DC	Dendritic cells
EC	Endothelial cells
ECM	Extracellular matrix
EGFR	Epidermal growth factor receptor
EMT	Epithelial to mesenchymal transition
FAP	Fibroblast-activation protein
FGF-2	Fibroblast growth factor-2
G-CSF	Granulocyte colony stimulating factor
HER2	Human epidermal growth factor receptor 2
HGF	Hepatocyte growth factor
HUVEC	Human umbilical vein endothelial cells
ICAM-1	Intercellular adhesion molecule 1
IFN-γ	Interferon- γ
IL-6	Interleukin-6
LOX	Lysyl oxidase
LPS	Lipopolysaccharide
MDSC	Myeloid-derived suppressor cells
MHC	Major histocompatibility complex
MMP	Matrix metalloproteinases
MSC	Mesenchymal stromal cells
M-CSF	Macrophage-colony stimulating factor
NK	Natural Killer cells
PBMC	Peripheral blood mononuclear cells
PEG	Poly(ethylene) glycol
PD-1	Programmed cell death protein 1
PD-L1	Programmed death ligand 1
PDGF	Platelet-derived growth factor
PMA	Phorbol-12-myristate-13-acetate
ROS	Reactive oxygen species
SMC	Smooth muscle cells
TAM	Tumour-associated macrophages
TGF-β	Transforming growth factor β
TIMP-1	Tissue inhibitor of metalloproteinases 1
TME	Tumour microenvironment
TNF-α	Tumour necrosis factor α
Treg	Regulatory T cells
VEGF	Vascular endothelial growth factor
α-SMA	α Smooth muscle actin
